# Long-Amplicon Single-Molecule Sequencing Reveals Novel, Trait-Associated Variants of *VERNALIZATION1* Homoeologs in Hexaploid Wheat

**DOI:** 10.3389/fpls.2022.942461

**Published:** 2022-07-15

**Authors:** Manar Makhoul, Harmeet S. Chawla, Benjamin Wittkop, Andreas Stahl, Kai Peter Voss-Fels, Holger Zetzsche, Rod J. Snowdon, Christian Obermeier

**Affiliations:** ^1^Department of Plant Breeding, Justus Liebig University Giessen, Giessen, Germany; ^2^Department of Plant Sciences, Crop Development Centre, University of Saskatchewan, Saskatoon, SK, Canada; ^3^Institute for Resistance Research and Stress Tolerance, Julius Kühn Institute, Quedlinburg, Germany; ^4^Institute for Grapevine Breeding, Hochschule Geisenheim University, Geisenheim, Germany

**Keywords:** wheat, vernalization, structural variation, copy number variation, haplotype, Oxford Nanopore Technologies

## Abstract

The gene *VERNALIZATION1* (*VRN1*) is a key controller of vernalization requirement in wheat. The genome of hexaploid wheat (*Triticum aestivum*) harbors three homoeologous *VRN1* loci on chromosomes 5A, 5B, and 5D. Structural sequence variants including small and large deletions and insertions and single nucleotide polymorphisms (SNPs) in the three homoeologous *VRN1* genes not only play an important role in the control of vernalization requirement, but also have been reported to be associated with other yield related traits of wheat. Here we used single-molecule sequencing of barcoded long-amplicons to assay the full-length sequences (∼13 kbp plus 700 bp from the promoter sequence) of the three homoeologous *VRN1* genes in a panel of 192 predominantly European winter wheat cultivars. Long read sequences revealed previously undetected duplications, insertions and single-nucleotide polymorphisms in the three homoeologous *VRN1* genes. All the polymorphisms were confirmed by Sanger sequencing. Sequence analysis showed the predominance of the winter alleles *vrn-A1*, *vrn-B1*, and *vrn-D1* across the investigated cultivars. Associations of SNPs and structural variations within the three *VRN1* genes with 20 economically relevant traits including yield, nodal root-angle index and quality related traits were evaluated at the levels of alleles, haplotypes, and copy number variants. Cultivars carrying structural variants within *VRN1* genes showed lower grain yield, protein yield and biomass compared to those with intact genes. Cultivars carrying a single *vrn-A1* copy and a unique haplotype with a high number of SNPs were found to have elevated grain yield, kernels per spike and kernels per m^2^ along with lower grain sedimentation values. In addition, we detected a novel SNP polymorphism within the G-quadruplex region of the promoter of *vrn-A1* that was associated with deeper roots in winter wheat. Our findings show that multiplex, single-molecule long-amplicon sequencing is a useful tool for detecting variants in target genes within large plant populations, and can be used to simultaneously assay sequence variants among target multiple gene homoeologs in polyploid crops. Numerous novel *VRN1* haplotypes and alleles were identified that showed significantly associations to economically important traits. These polymorphisms were converted into PCR or KASP assays for use in marker-assisted breeding.

## Introduction

For temperate crops like winter wheat, vernalization during a prolonged period of low winter temperature is required to induce transition from vegetative growth to flowering in spring. A key regulator involved in this transition process in cereal crops is the gene *VERNALIZATION1* (*VRN1*). *VRN1* has also been found to influence numerous other developmental processes and yield-related traits including plant height, spike and spikelet development, seed yield and frost tolerance (Babben et al., 2018; Li et al., 2019; Hyles et al., 2020). *VRN1* encodes a MADS-box transcription factor which promotes plant development and flowering by interacting with many downstream target genes including *VRN2*, *VRN3*, *FT1*, and *ODDSOC2* (Deng et al., 2015). Thus, detailed analysis of genetic polymorphisms in the key regulator gene *VRN1* and correlations with agronomic traits are of high interest for breeding to increase grain yield potential. Hexaploid bread wheat harbors three homoeologous copies of *VRN1* located on the 5A, 5B, and 5D chromosomes (Danyluk et al., 2003; Trevaskis et al., 2003; Yan et al., 2003; Cockram et al., 2007a). Illegitimate recombination plays an important role in the creation of novel alleles in *VRN1* conferring adaptation to annual cropping systems in barley and wheat (Cockram et al., 2007b). The dominant vernalization-insensitive spring alleles *Vrn-A1a* and *Vrn-A1b* on chromosome 5A are characterized by insertions, deletion and single nucleotide polymorphisms (SNPs) within the promoter regions (Yan et al., 2004; Muterko et al., 2016). The dominant vernalization-insensitive spring allele *Vrn-B1a* on chromosome 5B is characterized by a deletion of 6,850 bp within the first intron (Fu et al., 2005).

Copy number variation (CNV) of *VRN1* also has been found to be involved in flowering time and that wheat plants with an increased copy number of *vrn-A1* have an increased requirement for vernalization (Díaz et al., 2012). Würschum et al. (2015) showed that *vrn-A1* has 1 to 3 copies among cultivars from a global wheat panel, with allele frequencies depending on the geographic origin of the cultivars. However, analysis of the effects of *vrn-A1* copy number on heading date in the field revealed contrasting effects for cultivars originating from the United States and some European countries. Kippes et al. (2015) identified a fourth vernalization gene *Vrn-D4* located on chromosome 5D in common wheat of South Asian origin. This gene is considered to be a copy of the *Vrn-A1* gene on chromosome 5A. Recently, copy numbers for the dominant alleles of *VRN-A1* and recessive allele of *VRN-B1* have been detected in different species of wheat by Muterko and Salina, 2018, 2019, 2021 and Strejčková et al. (2021).

Rapid progress in the DNA sequencing technologies is providing valuable new insights into the genetic basis of traits. Short-read sequencing approaches like Illumina sequencing have been widely used for high-throughput sequencing with pooled, barcoded samples as an efficient approach for whole-genome resequencing (e.g., Marroni et al., 2012; Fritsch et al., 2016; Hawliczek et al., 2020). However, resolution of copy number variants or structural variants (SVs) can be difficult using short-read technologies, particularly in complex polyploid crop genomes where reads cannot always be unanimously assigned to a single homoeologous gene copy. In contrast, the increasing accuracy of long-read, sequencing approaches make them extremely useful for distinguishing between homoeologs, resolving haplotypes and detecting SVs in complex genomes (e.g., Sedlazeck et al., 2018; Mantere et al., 2019, Chawla et al., 2020). Multiplex approaches for long-read sequencing have been applied in virus and microbial community analyses (e.g., Quick et al., 2017; Calus et al., 2018; Liou et al., 2020), but have not yet been widely tested in plant populations. This study aims at an in-depth analysis of *VRN1* homoeologue sequence variability including structural variations (SVs), CNVs, and SNPs based on multiplex next-generation sequencing technology especially in winter wheat and analysis of their correlations with multiple agronomic traits. For this reason, we applied an approach based on Oxford Nanopore Technology (ONT) amplicon-based multiplex sequencing for simultaneous genetic analysis of homoeologous *VRN1* genes from 192 hexaploid bread wheat cultivars.

## Materials and Methods

### Phenotyping Data

The diversity panel used in this study comprised of 192 bread wheat cultivars, including 167 elite European winter wheat cultivars (Voss-Fels et al., 2019b) and 25 additional diverse cultivars from Europe, Chile, Mexico, United States, India and Australia (Lichthardt et al., 2020). Part of the phenotype data was published previously as part of a larger dataset for a collection of 191 wheat cultivars by Voss-Fels et al. (2019b). The phenotype data used in this study for a subset of 167 cultivars are provided again, plus phenotype data for 25 additional cultivars in Supplementary Table 1. Wheat cultivars were analyzed in field and laboratory experiments in Germany for grain yield [dt/ha], biomass [t/h], thousand kernel weight (TKW) [g], sedimentation value, falling number [seconds], kernels per spike, kernels per m^2^, spikes per m^2^, harvest index, plant height [cm], heading date, nitrogen use efficiency (NUE) [index], stripe rust infection [% of non-infected leaf area], powdery mildew infection [% of non-infected leaf area], crude protein [%], protein yield [kg/ha], radiation use efficiency (RUE) [g per MJ], radiation interception efficiency (RIE) and Green canopy duration (GCD) [°Cd] (Voss-Fels et al., 2019b). RUE is the ratio of above-ground biomass during the growing season to the sum of intercepted effective radiation. RIE is the ratio of total intercepted effective radiation to total effective radiation. Measurement and calculation of RUE and RIE are described in Rose and Kage (2019). GCD is the difference between the temperature at which the green leaf area drops to 50% and the thermal time at heading date. Measurement and calculation of GCD is described in Lichthardt et al. (2020). Phenotyping data represents adjusted means over six locations and two growing seasons for plots fertilized with 220 kg/ha N along with full intensity of fungicides, insecticides and growth regulators, representing standard agrochemical applications under intensive wheat production conditions in western Europe. Powdery mildew and stripe rust scores were recorded in both growing seasons only in the fungicide-free treatments. Phenotyping data for mean values of nodal root-angle index (NRI) for three independent greenhouse experiments was obtained from Supplementary Data 1 in Voss-Fels et al. (2018).

### DNA Extraction

Total genomic DNA was extracted from young leaf tissues using the BioSprint 96 DNA Plant kit (Qiagen, Düsseldorf, Germany) according to the manufacturer’s recommendations. DNA concentrations were quantified using the Qubit dsDNA BR Assay kit from Invitrogen and a microplate reader with fluorescence excitation/emission (TECAN infinite 200, Männedorf, Switzerland).

### Primer Design and PCR Amplification of *VERNALIZATION1*

A specific primer pair (VRN1F, VRN1R) targeting the entire full-length coding plus promoter sequence of three homoeologous *VRN1* loci on chromosome 5A, 5B, and 5D was developed manually based upon a multiple alignment of *VRN1* sequences previously published (including sequences published by the 10 + genome project; IPK Crop Analysis Tools Suite, 2021). The estimated product sizes ranged from 5 to 14 kbp depending on the gene copy and previously known SVs. The target-specific primers were tailed with universal sequences at 5‘ end to attach ONT barcodes in a second PCR reaction (primer sequences are available in Supplementary Table 2). The first-round PCR amplification was performed in 50 μl containing 16.5 μl RNase-free water, 25 μl of GoTaq Long PCR Master Mix, (Promega, Madison, WI, United States), 2.5 μl of each primer (10 μM), and 3.5 μl (60 ng/μl) genomic DNA. PCR reactions were performed in a T100 Thermal Cycler (Bio-Rad Laboratories, Hercules, CA, United States) using the following program: initial denaturing at 94°C for 2 min, followed by 35 cycles of 94°C for 30 s, 64°C annealing/extension for 13 min and 30 s, with a final extension step at 72°C for 10 min. Agarose gel electrophoresis was used to ensure PCR amplification success. Afterward, the PCR products were purified *via* AMPure XP beads (Beckman Coulter, Brea, CA, United States) to remove salts, primers, primer dimers and proteins. The second round of PCR amplification which aims to add barcode sequences into the amplicons was carried out in 50 μl reaction volumes consisting of 25 μl LongAmp *Taq* 2X Master Mix (New England Biolabs, City, United Kingdom), 24 μl of the first-round PCR products, and 1 μl of a barcode primer (EXP-PBC096, ONT, Oxford, United Kingdom). PCR conditions used for barcoding were as follows: an initial denaturing step at 95°C for 3 min, followed by 18 cycles of denaturation for 15 s at 95°C, annealing for 15 s at 62°C, and extension for 13 min and 40 s at 65°C, the final extension step was carried out at 65°C for 13 min. PCR was followed by purification of the amplicons using AMPure XP beads, DNA quantity was measured and equal amounts of all 96 samples were pooled into a single sequencing library.

### Oxford Nanopore Technology Library Preparation and Sequencing

The MinION library was produced using the Ligation Sequencing Kit 1D (SQK-LSK109, ONT Oxford, United Kingdom) according to the manufacturer’s recommendations, with the following modifications. DNA repair and end-prep reaction was incubated in a PCR thermocycler for 30 min at 20°C followed by 30 min at 65°C. About 28 fmol of pooled diluted library calculated from Qubit measurement was loaded and sequenced on MinION R9.4.1 flow cell for approximately 24 h, until no further sequencing reads could be collected. After the run was completed, the flow cell was washed with Flow Cell Wash Kit (EXP-WSH003, ONT, Oxford, United Kingdom), and was used again for resequencing of the same pooled library.

### Reference Based Alignment and Structural Variant Detection

The raw fast5 files obtained by the MinION instrument were processed using the base-caller Guppy version 4.5.4 + 66c1a77 with model “dna_r9.4.1_450bps_hac.cfg” (Oxford Nanopore Technologies, 2020). The guppy_barcoder was used to demultiplex the basecalled reads, with the option *detect_mid_strand_barcodes*. NanoStat version 1.5.0 was applied to assess the read quality and read statistics (De Coster et al., 2018). Reads with Q-score lower than 8, length less than 2,000 bp and more than 16 kbp were filtered out by using the NanoFilt v.2.8.0 tool (De Coster et al., 2018). Filtered reads were aligned to the *VRN1* sequences of Robigus scaffold as reference genome (Walkowiak et al., 2020; Earlham Institute, 2021) using the NGMLR long-read mapper version 0.2.7 (Sedlazeck et al., 2018), with setting min-identity 0.80 and min-residues 0.50. Subsequently, the alignment files in SAM format were converted to sorted BAM files, with map quality *q* > 50, and indexed using Samtools version 1.7 (Li et al., 2009). SVs were called by the software Sniffles version 1.0.12 (Sedlazeck et al., 2018), with option min_support 10. Afterward, the SURVIVOR v.1.0.7 merge tool was used to merge SV calls per barcode and compare overlaps among SV calls (Jeffares et al., 2017). The aligned reads and SVs were visually inspected by the Integrative Genomics Viewer IGV (Robinson et al., 2011).

### *De novo* Assembly and Consensus Sequence Generation From Oxford Nanopore Technology Data

Three different pipelines were applied to obtain full-length sequences of *VRN1* using ONT sequencing data (see scheme in Supplementary Figure 1). The *VRN1* sequences resulting from the three pipelines were aligned against a reference database constructed from publicly available wheat reference genome sequence data (IPK Crop Analysis Tools Suite, 2021) and complete *VRN1* gene sequences of up to 20 cultivars (NCBI, 2021) using ncbi-blast-2.6.0+ (Camacho et al., 2009). *VRN1* sequences showing identity values with the top blast hit of less than 99% were discarded from subsequent analyses.

### Single Nucleotide Polymorphisms Calling

Single nucleotide polymorphisms from read alignments stored within bam files were called using Medaka version 1.0.3 (Oxford Nanopore Technologies, 2021). BCFtools merge v1.9 (Danecek et al., 2021) and VCFtools 0.1.16 were used to merge multiple VCF files into one combined file and to filter out SNPs that had a minor allele count less than three and a minimum quality score of 30. Also, the tool SNP-sites (Page et al., 2016) was used to call SNPs from multi-alignment of consensus sequences aligning to a reference sequence with considering only genome regions present in a reference. To reduce the number of incorrect variant calls due to the high error rate of ONT (Rang et al., 2018; Delahaye and Nicolas, 2021), most of SNPs located in homopolymeric regions were excluded from downstream analysis. Detected SNPs and some indels were also visually inspected in the Integrative Genomics Viewer (Robinson et al., 2011) and compared to publicly available reference genomes. The QGRS mapper (Kikin et al., 2006) was used to analyze the G4 motif within the sequences of three homoeologous genes and their promoters using the pattern participating in the G4 structure formation (Capra et al., 2010; Williams et al., 2020).

### Sanger Sequencing and Gel Electrophoresis

To validate SVs and SNPs detected in *VRN1* genes, 12 locus-specific primer pairs were developed using the program Primer3 v0.4.0 (Untergasser et al., 2012) to amplify the target regions (primer sequences are listed in Supplementary Table 2). PCR amplification was performed in 25 μl containing 8.5 μl RNase-free water, 12.5 μl of GoTaq Hot Start Colorless Master Mix, (Promega, Madison, WI, United States), 1.25 μl of each primer (10 μM), and 1.5 μl (60 ng/μl) genomic DNA. PCR reactions were performed in a T100 Thermal Cycler (Bio-Rad Laboratories, Hercules, CA, United States) using the following program: initial denaturing at 95°C for 2 min, followed by 35 cycles of denaturation at 94°C for 30 s, annealing temperature at 58–65°C (the Ta value depends on the structure of primers and of the amplified region) for 35 s, and extension at 72°C for 45 s up to 7 min (the extension time depends on the length of the sequence to be amplified, usually 1 min for each kbp), with a final extension step at 72°C for 10 mins. Agarose gel electrophoresis was used to separate fragments of PCR products. Sanger sequencing was performed from one or both directions using the same forward and reverse primers applied for PCR amplification by Microsynth Seqlab GmbH (Göttingen, Germany).

### KASP Markers

Eight KASP markers were developed from the identified polymorphisms (Supplementary Table 3). The KASP assay procedure was performed following the method outlined in Makhoul et al. (2020). All PCR primers were synthesized at Microsynth Seqlab (Göttingen, Germany) with desalting purification. To obtain distinct clear genotyping clusters from VRN5A-SNP45K KASP marker, we used PCR products resulting from pre-amplification of the SNP45 flanking region instead of genomic DNA in a KASP reaction mixture (Makhoul and Obermeier, 2022).

### Statistical Analyses

Statistical analysis was carried out using R software version 4.1 (R Core Team, 2021). The Tukey Honest Significant Difference (Tukey HSD) test was used to make pairwise comparison of means for a set of groups, using the R package agricolae (De Mendiburu, 2021). To estimate statistical significance for differences between two groups, the non-parametric Wilcoxon rank-sum test (Wilcoxon, 1945) and parametric Student’s *t*-test and Welch’s *t*-test were applied (Student, 1908; Welch, 1947). The differences at *p*-value ≤ 0.05 were considered to be significant.

## Results

### An Amplicon-Based Multiplex Oxford Nanopore Technology Approach Allows Efficient Sequence Analysis of Homoeologs of the *VERNALIZATION1* Gene Within a Wheat Population

PCR fragments of the gene copies *VRN-A1*, *VRN-B1*, and *VRN-D1* with a total length of up to 14 kbp per amplicon were simultaneously amplified using a single primer pair, and multiplexed long-read sequencing libraries were successfully generated using a second round of PCR to attach 96 ONT-compatible barcodes followed by ONT library preparation. All *VRN1* gene copies were sequenced from a total of 192 wheat cultivars using two ONT MinION flow cells, producing at total of 11.2 Gb and 14.7 million reads. About 34% (3.8 Gb) of the total data could not be assigned to the barcoded libraries (cultivars) due to a lack of assignable barcode sequences (unclassified reads, Supplementary Figure 2A). About 15.2% (1.7 Gb, 4.2 million reads) of the total data were predicted to be chimeric by the software Guppy, due to barcode sequences located in the middle of reads. Furthermore, data analysis revealed that 4,256 reads were longer than 16 kbp, of which 62% (2,635 reads) were classified as chimeric. After elimination of chimeric and unclassified reads, around 5.7 Gb and 7.1 million reads remained in the respective libraries. Finally, after trimming and filtering for reads with length < 2 kbp or >16 kbp, and a quality score < 7, approximately 3.54 Gb and 641k reads with average length of 6 kbp, were left for downstream analysis (Supplementary Figure 2A). The minimum and maximum data output for the 192 cultivars were 4.7 Mb and 63 Mb, respectively, with a mean of 34 Mb (±29 standard deviation, SD). Only uniquely mapping reads were considered after mapping of these filtered 641K reads against the Robigus *VRN1* gene reference consisting of the A, B, and D homoeologous genes. Here, Robigus was used as a reference because it carries the recessive winter alleles (intact alleles) for the three homoeologous *VRN1* genes. In total, 261k reads (1.77 Gb) were aligned to one of the three homoeologous *VRN1* loci, of which 114k reads (755 Mb) aligned to *VRN-A1*, 62.4k reads (399 Mb) aligned to *VRN-B1*, and 84.8k reads (618 Mb) aligned to *VRN-D1*, (42.5% *VRN-A1*, 22.5% *VRN-B1*, 35% *VRN-D1*).

Some cultivars showed extremely low or high coverage for at least one of the three homoeologous *VRN1* genes. The low-coverage outliers consisted of the cultivar Atomic and the cultivars Kraka, Tommi, Hybery, Sonalika and the cultivars Highbury, Lambriego Inia, Triple Dirk S and INTRO 615, with a coverage less than 11x for *VRN-A1*, *VRN-B1*, and *VRN-D1*, respectively. *VRN-A1* showed the highest variability in coverage across all cultivars, with a coefficient of variation (CV) of 82% compared to 61% CV for *VRN-B1* and 45% CV for *VRN-D1*. The high variance in *VRN-A1* may be due to copy-number variation in *VRN-A1* within the wheat haploid genome across cultivars, as described before by Díaz et al. (2012); Würschum et al. (2015); Muterko and Salina (2019). We also found that there is an uneven sequencing depth across the three homoeologous *VRN1* genes within different barcoded libraries (cultivars). The ratio of total bases between *VRN-A1*/*VRN-B1* ranged from 0.02 to 5,250 (median = 2), and from 0.00051 to 12,047 (median = 0.50) for *VRN-B1*/*VRN-D1*, while for the *VRN-A1*/*VRN-D1* ratio it ranged from 0.1 to 411 (median = 1.4). This variance between *VRN1* genes within cultivars might be due to differences in PCR amplification efficiency within one cultivar for the A, B, and D homoeologous genes (e.g., due to sequence divergence in priming sites, GC-poor or GC-rich sequences and amplification of a mixture of PCR products of variable length). For four of the outlier cultivars, it was confirmed that one homoeologous copy contains a large deletion. For example, the cultivar Highbury harbors a large deletion of 6,851 bp within the first intron of gene *VRN-B1*. This deletion putatively led to preferential PCR amplification of *VRN-B1* (small amplicon) relative to *VRN-A1* and *VRN-D1* (large amplicons), resulting in an extremely high coverage of 4,023× for *VRN-B1*, in contrast to 0.17× and 41× coverage for *VRN-D1* and *VRN-A1*, respectively. For the outlier cultivar Sonalika, which had low coverage of 10.7× for *VRN-B1*, Sanger sequencing showed one mismatch (A/C) at position 8 upstream of the 3’ end of the primer binding site for the forward primer (VRN1F), potentially causing a partial failure of PCR amplification. The mean coverage of each *VRN1* locus after removal of extreme outlier cultivars (for detailed information see Supplementary Table 4) from the dataset showed the highest mean coverage for *VRN-A1* with 306× (±250 SD), followed by *VRN-D1* with 258× (±115 SD) and *VRN-B1* with 133× (±81 SD; Supplementary Figure 2B). However, in spite of these coverage variations, a sufficient amount of data was still produced to enable accurate population-wide analyses. Thus, our approach based on using a single primer set to simultaneously amplify all homoeologs represents an acceptable and cost-effective way to avoid the use of three or more pairs of homoeolog-specific primer sets for single-molecule amplicon library production.

### Dissection of Homoeologs Requires a Combination of *de novo* Assembly and Reference-Based Alignment

Three different customized pipelines were used to create and evaluate consensus sequences from ONT sequence data. In the first pipeline (A_denovo), a *de novo* assembly approach was undertaken to avoid generating any bias due to the used reference sequence, while in the other two pipelines (B_align_denovo, C_align), the reads were initially or exclusively mapped to a reference sequence to generate homoeologue consensus sequences using two different approaches (see Supplementary Figure 1). Applying a *de novo* assembly strategy in the A_denovo pipeline without mapping the reads to a reference allowed us to identify an additional contig resulting from amplification of a non-target region on chromosome 4A (resulting from partial annealing of *VRN1* primers). Due to the high similarity between the three homoeologous *VRN1* sequences (percentage of identity is approximately 92%), the A_denovo pipeline resulted in the assembly of many erroneous unrelated sequences and generated more fragmented contigs. Pipeline B is less sensitive to *de novo* assembly artifacts based on high similarity of homoeologous sequences. However, comparison of results of pipeline A with B and C allows to identify translocations and insertions/deletions and examine the accuracy of the produced consensus sequence. Pipeline C_alignments also allow to obtain consensus sequences for cultivars with low coverage. Adding a polishing step can also improve the accuracy of most consensus sequences by reducing the number of indels and correcting errors, but also it caused sometimes other errors for some cultivars in some situations.

### *VERNALIZATION1* Homoeolog Combinations in Analyzed Cultivars Are Identical to Only Five Reference Genomes

**FIGURE 1:**
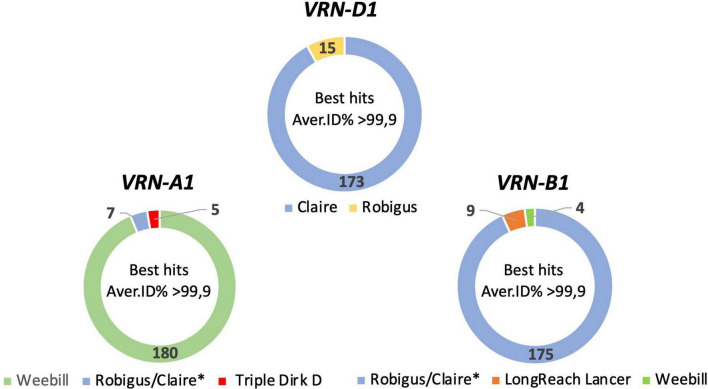
Pie graphs showing the classification for full-length *VRN1* sequences from 192 wheat cultivars based on the result of top BLASTn hits against a *VRN1* reference database (average > 99.9% identity). The numbers represent the number of cultivars in each group. *The *VRN-A1* and *VRN-B1* sequences are identical in references genomes Claire and Robigus. The *VRN-D1* sequences are different in reference genomes Claire and Robigus.

Based on the combined results of all three pipelines 192, 188, and 188 full length sequences of *VRN-A1*, *VRN-B1*, and *VRN-D1* genes were obtained. These full-length sequences showed a best hit identity in BLASTn analysis of 99.2 to 100% compared to the *VRN1* genes of only five reference genomes (see Supplementary Tables 5–7 for further information). For eight other wheat cultivars it was not possible to generate full-length consensus sequences of *VRN-B1* or *VRN-D1* due to low coverage for both genes. According to the top BLASTn hit outputs, the *VRN-A1* sequences were classified into three categories: *VRN-A1* Weebill type sequence, Robigus/Claire type sequence and Triple Dirk D type sequence (Figure 1). *VRN-B1* consensus sequences were classified into three categories: *VRN-B1* Robigus/Claire type sequence, LongReach Lancer type sequence and Weebill type sequence. *VRN-D1* sequences were classified into two categories: *VRN-D1* Claire type sequence and Robigus type sequence. Based on the combination of full-length sequence types for the three *VRN1* homoeologue sequences for each cultivar, the wheat cultivars were classified into nine sequence type groups as shown in Table 1. More than 83% of the studied cultivars carried a *VRN-A1*-Weebill type sequence and a *VRN-B1*-Robigus/Claire type sequence combined with a *VRN-D1*-Claire type sequence (detailed information in Supplementary Tables 8, 9). The reason why the blast best hit showed highest similarity with only five references is due to incompleteness of *VRN1* sequences in some of the available reference genomes (e.g., the *VRN-A1* sequences for ArinaLrFor, Mattis, and Julius references are fragmented into two or three small pieces located on unknown chromosomes).

**TABLE 1:** Grouping of 184 wheat cultivars based on top BLASTn hits of full-length *VRN1* sequences against around 30 reference genomes reveal nine groups of A, B, and D homeolog sequence combinations most similar (>99.9%) to only five reference genomes.

*VRN1* sequence type	*VRN-A1* best full-length hit with reference	*VRN-B1* best full-length hit with reference	*VRN-D1* best full-length hit with reference	Number of cultivars
S1	Weebill	Robigus/Claire*	Claire	154
S2	Robigus/Claire*	Robigus/Claire	Claire	4
S3	Weebill	Robigus/Claire	Robigus	13
S4	Weebill	LongReach Lancer	Claire	7
S5	Triple Dirk D	Robigus/Claire	Claire	1
S6	Triple Dirk D	LongReach Lancer	Claire	1
S7	Weebill	Weebill	Claire	1
S8	Triple Dirk D	Robigus/Claire	Claire	1
S9	Robigus/Claire	Robigus/Claire	Robigus	2
				184

*The VRN-A1 and VRN-B1 sequences are identical in references genomes of Claire and Robigus.

### Known and Novel Structural Variants Were Identified in Some Wheat Cultivars

**FIGURE 2:**
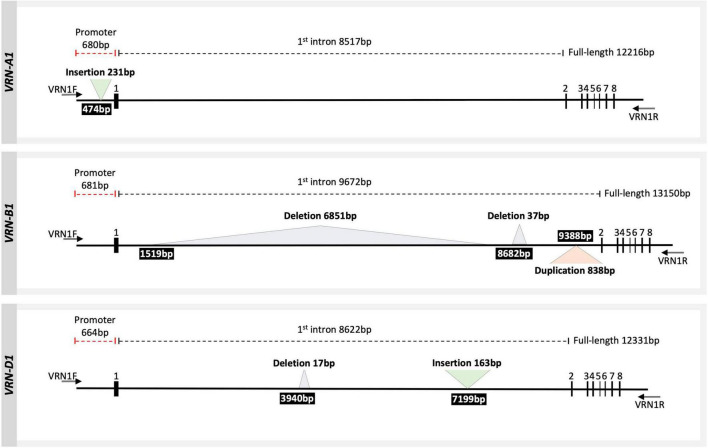
Scheme of three homoeologous wheat *VRN1* genes including complete intron–exon regions and partial promoter region. Exons are represented by numbers above bold boxes. The numbers above the dashed line represent the region length according to the Weebill, Robigus, and Claire reference sequences for *VRN-A1*, *VRN-B1*, and *VRN-D1*, respectively. The triangles represent the SVs detected in this study. The number within the black box indicates the start position of structural variation SV in a sequence (counting from the 3′ end of the forward primer VRN1F). The two arrows indicate the positions of primers used to amplify the full-length sequence of the three homoeologous *VRN1* genes (VRN1F and VRN1R).

The *VRN1* alleles have been classified in the literature into dominant spring alleles and into recessive winter alleles based on structural sequence variations. An insertion or a deletion in the promoter or within the first intron has a strong effect on the vernalization requirement, leading to a spring growth habit or a decreased requirement for vernalization (Yan et al., 2004; Fu et al., 2005; Shcherban et al., 2012; Muterko et al., 2015). We identified five SVs larger than 30 bp across the three homoeologous *VRN1* loci using long sequencing reads in 15 diverse cultivars from Europe, Chile, Mexico, United States, and Australia (Figure 2 and Table 2). In *VRN-A1*, an insertion of a foldback repetitive element of 231 bp was detected within the promoter region in five cultivars (Table 2). This insertion was similar to the one found in the dominant (spring) *Vrn-A1a* allele (Yan et al., 2004). In *VRN-B1*, three types of SV were detected. Firstly, a large deletion of 6851 bp was detected within the first intron region of four cultivars. This allele has been detected before and has been designated as dominant (spring) *Vrn-B1a* allele by Fu et al. (2005). Secondly, a 37 bp deletion located downstream of the large deletion within the first intron was detected in one cultivar (INTRO 615). This allele was referred to as dominant *Vrn-B1b* allele by Santra et al. (2009). Thirdly, a duplication of 838 bp within the first intron region was found in nine wheat cultivars. The same duplication was also observed in the reference genome of LongReach Lancer (see Supplementary Figure 3). Recently, this duplication was detected in three spring wheat genotypes and referred to as *Vrn-B1f* by Strejčková et al. (2021). In *VRN-D1*, a novel allele with a 163 bp insertion in the first intron was identified in two cultivars, Mex. 3 and Mex. 17 bb (hereafter referred to as *Vrn-D1x*; Supplementary Figure 4). In addition, a 17 bp deletion was detected in the first intron of *VRN-D1* in 15 cultivars (hereafter referred to as *vrn-D1r*). The same deletion also was found in the reference genome for Robigus (Supplementary Figure 5). The authenticity of all SVs identified from ONT reads were confirmed by Sanger sequencing and PCR amplification, followed by agarose gel electrophoresis using a set of primers listed in Supplementary Table 2. SV analysis showed that 4 out of 5 cultivars harboring the dominant *Vrn-A1a* spring allele combine with other dominant alleles at either *VRN-B1* or *VRN-D1* genes, or both (Table 2), supporting previous studies which found that spring wheat carries the dominant *Vrn-A1a* allele, either alone or in combination with the other dominant alleles at the *VRN-B1* locus (Goncharov and Shitova, 1999; Shcherban et al., 2012). As expected, we did not detect any known dominant spring alleles at the three *VRN1* loci in the subset of the 167 elite winter wheat cultivars released within the last 50 years in Europe (Voss-Fels et al., 2019b).

**TABLE 2:** Cultivars with structural variations larger than 30 bp at three *VRN1* loci.

	*VRN-A1*		*VRN-B1*		*VRN-D1*	
			
Cultivar name	Reported growth habit	Insertion (231 bp)	Duplication (838 bp)	Deletion (6,851 bp)	Deletion (37 bp)	Insertion (163 bp)
Hope*	S^1,2^	*Vrn-A1a*	–	–	–	–
Mex. 17 bb*	S^1^,W^2^	*Vrn-A1a*	–	–	–	*Vrn-D1x*
Mex. 3*	S^1,2^	*Vrn-A1a*	*Vrn-B1f*	–	–	*Vrn-D1x*
Highbury*	S^1,2^	*Vrn-A1a*	–	*Vrn-B1a*	–	–
INTRO 615*	W^2^	*Vrn-A1a*	–	*Vrn-B1a*	*Vrn-B1b*	–
Cappelle Desprez*	W^1,2^	–	–	*Vrn-B1a*	–	–
NS 22/92*	W^2^	–	–	*Vrn-B1a*	–	–
Joss	W^1,2^	–	*Vrn-B1f*	–	–	–
Tambor	W^1,2^	–	*Vrn-B1f*	–	–	–
NaturaStar	W^1,2^	–	*Vrn-B1f*	–	–	–
Topfit	W^1,2^	–	*Vrn-B1f*	–	–	–
Phoenix	W^1,2^	–	*Vrn-B1f*	–	–	–
Triple Dirk S*	S^2^	–	*Vrn-B1f*	–	–	–
Cajeme 71*	S^1,2^	–	*Vrn-B1f*	–	–	–
Lambriego Inia*	S^1^,W^2^	–	*Vrn-B1f*	–	–	–

*S, Spring growth habit; W, Winter growth habit.*

*^1^Genetic Resources Information System for Wheat and Triticale (2022).*

*^2^Gerard et al., 2018.*

*These cultivars were considered as spring wheat.

### Single Nucleotide Polymorphism Calling Revealed Six Genotype Groups for *VRN-A1*

Based on the full-length sequence similarity of *VRN-A1* for the 192 cultivars to reference genomes the cultivars can be divided into three *VRN-A1* sequence types, Triple Dirk D, Robigus/Claire, and Weebill (Table 3). For accurate SNP calling we applied strict filtering parameters to eliminate false calls which might be expected due to high error rates of ONT sequencing (Menegon et al., 2017). We identified 48 polymorphisms within the complete *VRN-A1* gene and three polymorphisms within the 700 bp of the promoter region. Of these 51 polymorphisms, 49 were also present in reference genomes. Nine detected SNPs were validated and confirmed either by Sanger sequencing or KASP markers in 192 cultivars (Table 3). KASP markers revealed mostly consistent results. However, two known hybrid cultivars were classified as heterozygous for most detected SNP loci (Hyland and Hybery). These two hybrid cultivars were excluded from downstream analysis. Detected high-confidence SNPs were mostly located within the first intron region of *VRN-A1*. This was expected due to its relatively large size (accounts for 70% of amplified target size). The first intron also has been considered a critical region in *VRN1* due to presence of putative regulatory elements (Fu et al., 2005; Distelfeld et al., 2009; Xiao et al., 2014; Muterko et al., 2015; Kippes et al., 2018). Based on SNP calls the cultivars could be further classified into six genotype groups (Table 3). Most cultivars belong to genotype group GT4 (111) and GT6 (55) which all have sequences very similar to the reference Weebill. However, 171 of these 179 cultivars in the Weebill *VRN1* sequence type group show for some SNPs heterozygous calls (positions 381, 4,138, 4,757, and 11,109). Genotype group GT1 including five cultivars which carry the dominant *Vrn-A1a* allele can be assigned to haplotype group Hap1, genotype group GT2 including 6 cultivars can be assigned to haplotype group Hap2 and genotype group GT3 can be assigned to haplotype group Hap3. For 171 cultivars (genotype groups GT4, GT5, and GT6) no haplotype group could be assigned due to heterozygous calls.

**TABLE 3:** Sequence groups, genotype groups, haplotypes, and SNPs detected in *VRN-A1* of 190 wheat cultivars^1^.

																																																						
			Region	Promoter			Intron 1										RIP-3†		Intron 1																											Intron 2		Exon 4	Intron 4				Intron 6	Exon 7
												
			Position^Φ^	147	244	381	1,781	1,890	2,066	3,184	3,296	3,298	3,386	3,393	3,408	3,431	3,459	3,463	3,701	3,999	4,138	4,646	4,703	4,740	4,757	5,270	5,634	5,722	5,801	5,928	5,932	6,129	6,160	6,568	6,907	6,978	7,468	7,625	7,738	8,036	8,201	8,266	9,062	9,736	10,201	10,802	10,909	11,109	11,234	11,235	11,236	11,344	11,639	11,735
												
*VRN-A1* sequence type	Genotype groups	Number of cultivars	Haplotypes^+^	SNP1*	SNP2*	SNP3*	SNP4	SNP5*	SNP6	SNP7	SNP8	SNP9	SNP10	SNP11	SNP12	SNP13	SNP14	SNP15	SNP16	SNP17	SNP18*	SNP19	SNP20	SNP21	SNP22*	SNP23	SNP24	SNP25	SNP26	SNP27	SNP28	SNP29	SNP30	SNP31	SNP32	SNP33	SNP34	SNP35*	SNP36	SNP37	SNP38	SNP39	SNP40	SNP41	SNP42	SNP43*	SNP44*	SNP45*	SNP46*	SNP47*	SNP48*	SNP49*	SNP50	SNP51*
																																																						
Triple Dirk D	GT1	5	Hap1	A	T	C	T	A	A	C	T	G	C	C	C	G	G	C	A	T	G	C	C	T	G	G	G	C	G	C	A	A	A	G	C	C	C	C	T	T	C	T	T	A	T	T	T	C	G	–	–	–	A	C
Robigus/Claire	GT2	6	Hap2	A	G	C	T	T	G	C	C	T	T	T	T	G	C	T	G	G	G	T	T	T	G	G	A	G	T	C	G	G	A	T	C	T	G	C	G	C	C	C	T	G	C	G	C	C	A	A	C	–	A	C
Weebill	GT3	8	Hap3	G	G	C	–	A	A	G	T	G	C	C	T	A	G	C	G	G	G	C	C	G	G	T	G	G	G	G	A	G	T	T	T	C	G	A	G	C	G	C	G	G	C	G	T	C	A	A	C	A	–	T
Weebill	GT4	111	Hap3, Hap4	G	G	C	–	A	A	G	T	G	C	C	T	A	G	C	G	G	G	C	C	G	G	T	G	G	G	G	A	G	T	T	T	C	G	A	G	C	G	C	G	G	C	G	T	**Y**	A	A	C	A	–	T
Weebill	GT5	5	Hap3, Hap4, Hap5, Hap6	G	G	**S**	–	A	A	G	T	G	C	C	T	A	G	C	G	G	G	C	C	G	**R**	T	G	G	G	G	A	G	T	T	T	C	G	A	G	C	G	C	G	G	C	G	T	**Y**	A	A	C	A	–	T
Weebill	GT6	55	Hap3, Hap4, Hap7, Hap8	G	G	C	–	A	A	G	T	G	C	C	T	A	G	C	G	G	**R**	C	C	G	G	T	G	G	G	G	A	G	T	T	T	C	G	A	G	C	G	C	G	G	C	G	T	**Y**	A	A	C	A	–	T

*^1^Two hybrid cultivars, Hybery and Hyland, were removed from analysis due to heterozygosity.*

*† RIP-3 is a putative binding site for the flowering repressor TaGRP2 (Xiao et al., 2014; Kippes et al., 2015).*

*^Φ^Positions of SNPs based on the Weebill reference sequence (counting from the 3′ end of the forward primer VRN1F).*

*^+^Resolution of haplotypes based on ONT phasing, KASP, Sanger sequencing.*

*SNP genotyping calls were validated with Sanger sequencing and KASP markers, bold and underlined letter represents heterozygous SNP.

### Haplotype Resolution Based on Oxford Nanopore Technology Long Reads

**FIGURE 3:**
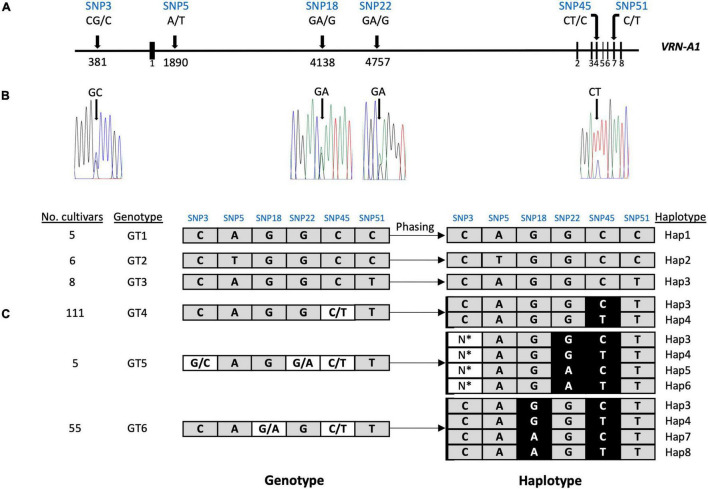
Phasing of the *VRN-A1* haplotypes using ONT long reads. **(A)** scheme shows the location of six SNPs in *VRN-A1*. **(B)** Four SNPs with overlapping peaks were detected in Sanger sequencing chromatograms. **(C)** Haplotypes were deduced from genotypes using ONT long reads. *Due to the SNP3 location between Gs, Cs repeats region and the presence of many indels in this region, it was difficult to identify the allele accurately from ONT reads.

We detected four SNPs (SNP3, SNP18, SNP22, and SNP45) showing heterozygous alleles in genotype groups GT4, GT5, and GT6 (Table 3). To exclude the possibility that false variant calls were resulting from misalignment of ONT reads to the reference genome and/or differences in coverage between allelic reads, we validated the genotyping calls of these SNPs by Sanger sequencing and KASP markers (Figures 3A,B and Supplementary Figures 6–9). Heterozygous calls in some cultivars could either indicate the presence of multiple copies of *VRN-A1* in the genome or the presence of a heterozygous *VRN-A1* copy. Because wheat is generally autogamous, the most likely explanation is that multiple copies of *VRN-A1* are present in the inbred lines investigated here. For genotype group GT4 harboring one heterozygous SNP45 within *VRN-A1*, KASP marker analysis and ONT analysis revealed that 111 cultivars carried two haplotypes, Hap3 and Hap4. For genotype groups GT6 and GT5 harboring 2 and 3 heterozygous SNPs within *VRN-A1*, respectively, we used ONT long reads overlapping the heterozygous SNPs to extract the information from genotype data by phasing the haplotypes (Figure 3C). The results showed that 54 out of 55 cultivars classified as genotype group GT6 harbored four haplotypes of *VRN-A1* (Hap3, Hap4, Hap7, Hap8, whereby one cultivar showed very low number of reads supporting each haplotype), and five cultivars classified as genotype group GT5 showed at least 4 haplotypes (Hap3, Hap4, Hap5, and Hap6). We were not able to accurately identify the allele of SNP3 based on ONT reads because this SNP is located between G and C repeats which cannot be clearly resolved by ONT sequencing.

### *VRN-A1* Copy Number Variation Validated by KASP Assays and Sanger Sequencing

**FIGURE 4:**
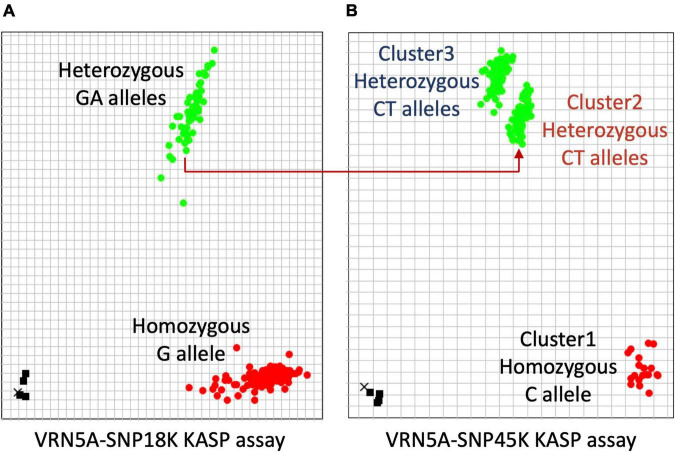
Two genotyping plots of KASP assays used for identifying the copy number of *VRN-A1*. **(A)** Plot of KASP assay for SNP18 in the first intron shows that the cultivars carrying the genotype group GT6 (four haplotypes/four copies) were assigned to the heterozygous cluster GA. **(B)** Plot of KASP assay for SNP45 in exon 4 shows that all cultivars containing three copies of *VRN-A1* were assigned to the heterozygous Cluster3.

Previous studies reported that the presence of the C allele at SNP within exon 7 (designated SNP51 in this study) in winter bread wheat is associated with a single copy of *VRN-A1*, while multiple copies of *VRN-A1* in wheat haploid genomes are related with the presence of a mutated T allele at SNP51 in exon 7 or the presence of a heterozygous SNP (C/T) in exon 4 (designated SNP45 in this study; Díaz et al., 2012; Würschum et al., 2015; Grogan et al., 2016; Muterko and Salina, 2018; Dixon et al., 2019). In this study, we detected four heterozygous SNPs in *VRN-A1* for 171 cultivars (Table 3). SNP18 located in intron 1 and SNP45 located in exon 4 showed an unexpectedly high frequency of heterozygous calls across cultivars, 29 and 90%, respectively. We developed two robust KASP markers (VRN5A-SNP18K and VRN5A-SNP45K) targeting these two SNPs within the *VRN-A1* gene. Surprisingly, the cluster plot of VRN5A-SNP45K KASP assay in exon 4 showed two distinct heterozygous clusters (Figure 4B). Cluster 2 (CT) is including all 55 cultivars carrying the combination of two alleles G/A at SNP18 (Figures 4A,B), while cluster 3 (CT) includes 107 cultivars carrying the G allele at SNP18 (Figures 4A,B). Based on this comparison we hypothesized that a dosage effect (gene copy number effect) for the T and C allele at SNP45 within exon 4 could be responsible for the formation of two distinct heterozygous clusters for the VRN5A-SNP45K assay. To investigate the allelic composition at SNP45, we computed the frequency of the mapped ONT reads supporting each allele using Samtools_mpileup tool. The results showed that the frequency of the T allele in cultivars of cluster 3 (∼70% in average) is higher than in those of cluster 2 (∼50% in average), with a strong significant difference (*P*-value > 2.2e-16). Based on this finding we hypothesize that cultivars of cluster 3 (CT) contain a higher number of *VRN-A1* copies with the T allele than those copies with the C allele. This current finding is supported by previous studies that have found different proportions of T and C alleles in exon 4 in hexaploid wheat based on end-point relative quantification of PCR fragments and exome-capture sequencing methods (Dixon et al., 2019; Muterko and Salina, 2019). To validate this hypothesis, we compared the copy number of *VRN-A1* of 122 winter wheat cultivars which had been previously reported and identified using TaqMan CNV assay by Würschum et al. (2015) with our findings. We found that 71 out of 72 cultivars reported to carry three copies were grouped in the cluster 3 (CT), while 43 out of 50 cultivars reported to carry two copies were grouped in cluster 2 (CT; detailed information in Supplementary Table 11). Comparison of the number of *VRN-A1* copies estimated by Würschum et al. (2015) by TaqMan assay qPCR with the number of haplotypes extracted from long reads in this study, revealed in contradiction that most cultivars with three copies contained two haplotypes, while the vast majority of cultivars with two copies contained four haplotypes (Table 4). Díaz et al. (2012) and other authors including (Zhu et al., 2014; Kippes et al., 2015; Würschum et al., 2015; Guedira et al., 2016; Dixon et al., 2019; Muterko and Salina, 2019; Dreisigacker et al., 2021; Strejčková et al., 2021) measured average fold-change ratio between the target gene *VRN-A1* relative to the internal positive control gene *CONSTANS2* (probes and primers binding to three homoeologous genes on 6A, 6B, and 6D). Thus, a ratio of 0.33 would represent one copy, 0.66 would represent two copies, 1.0 would represent 3 copies, and 1.3 would represent four copies. However, some of the studies assigned 2 haploid copies from a relative ratio of 0.7–0.9, while they assigned three haploid copies from a relative ratio of 1.1–1.3, instead of 4 haploid copies (Zhu et al., 2014), while other authors use different ranges to assign copy numbers. *CONSTANS2*, a flowering time regulator gene with a central role in plant growth and development, should not be considered an internal positive control gene, as in the absence of any functional copies of *CONSTANS2* heading time is controlled by *Photoperiod 1*, *CONSTANS1*, and *CONSTANS2* may thus also be affected by CNV in wheat (Shaw et al., 2020). *CONSTANS*-like genes in cereals are known to be affected by CNV (Cockram et al., 2010). In contrast, assignment of copy numbers by haplotypes allows to estimate a minimum number of present copies more accurately (Table 4). However, to estimate the exact copy number a combination of methods is required. E.g., frequency analysis of ONT allelic reads together with KASP analysis revealed two and three copies for GT4 while ONT haplotype analysis alone revealed only two.

**TABLE 4:** Comparison of copy number estimates for *VRN-A1* for 166 cultivars based on different methods.

Genotype group	Number of cultivars	Haplotypes	Haplotype number (minimum estimated copy number)	Cultivars cluster on VRN5A-SNP45K KASP plot	Average of allele frequency at SNP45 in exon 4 of *VRN-A1* from ONT reads	Copy number estimated based on haplotype number, allele frequency, KASP cluster	Copy number *VRN-A1* estimated by TaqMan qPCR (Würschum et al., 2015)
GT4	102	Hap3, Hap4	2	Cluster3	T: 70%, C:%30	3	3^A^
GT4	9	Hap3, Hap4	2	Cluster2	T: 51%, C:%49	2	2^B^
GT6	55	Hap3, Hap4, Hap7, Hap8	4	Cluster2	T: 51%, C:%49	4	2^C^

*^A^For 71 out of 75 cultivars common with this study Würschum et al. (2015) estimated three copies.*

*^B^For 5 out of 5 cultivars common with this study Würschum et al. (2015) estimated two copies.*

*^C^For 38 out of 39 cultivars common with this study Würschum et al. (2015) estimated two copies.*

### *VRN-B1* Sequence Analysis Revealed Five Haplotypes

Most SNPs called from *VRN-B1* ONT sequences were located in homopolymeric regions and/or these SNPs were present in fewer than three cultivars, suggesting that they are not highly reliable. Six SNPs were detected in *VRN-B1* from ONT data (Table 5). One SNP is located within intron 2, while the other five SNPs are located in intron 1. Two SNPs at positions 9,080 and 9,387 in intron 1 are heterozygous due to single nucleotide differences between nearly identical tandemly duplicated sequence segments of 838 bp. Based on SNPs and SVs detected in *VRN-B1* sequences, the investigated cultivars classified into five haplotype groups as shown in Table 5. Hap1 group includes the majority of 163 cultivars carrying the winter allele, while Hap4 group containing 11 cultivars was distinguished from Hap1 with a single novel SNP at position 8,615 bp in intron 1 (Supplementary Figure 10). The cultivars Benni multifloret (United States) and Sonalika (India) which showed many different polymorphisms within promoter and intron 1 were assigned to Hap3. The cultivars containing a duplication of 838 bp and a deletion of 6,851 bp in their first introns were grouped in Hap2 and Hap5, respectively.

**TABLE 5:** SNPs and haplotypes detected in *VRN-B1* genes of 188 cultivars^1^.

	Position^Φ^	7,505	8,351	8,615*	9,080	9,387*	10,971*

Haplotypes	Number of cultivars	Intron 1					Intron 2
Hap1	163	G	G	G	T	A	G
Hap2	9	A	A	G	T|C†	A|C**†**	A
Hap3	2	G	G	G	T	A	A
Hap4	11	G	G	A	T	A	G
Hap5	4	–	–	G	T	A	G

*^1^Four cultivars are not included the table due to their low coverage (Tommi, Kraka) and because they are hybrids (Hybery and Hyland).*

*^Φ^Position of SNPs is based on the Claire reference sequence (counting from the 3′ end of the forward primer VRN1F).*

*SNP genotyping was validated with KASP markers and Sanger sequencing.

*† Heterozygous calls T/C and A/C at these positions are due to single nucleotide differences between nearly identical duplicated sequence segments of 838 bp in VRN-B1.*

### *VRN-D1* Sequence Analysis Revealed Low Sequence Variation

*VRN-D1* sequences showed less variation across the cultivars in comparison with *VRN-A1* and *VRN-B1* sequences. In addition to the insertion (163 bp) and deletion (17 bp) discovered in the first intron of 2 and 15 cultivars, respectively, only one SNP (A/G) was identified at position 5,607 bp in the first intron of four cultivars (counting from the 3’ end of the forward primer VRN1F based on Claire reference sequence). Four haplotype groups were generated for *VRN-D1* sequences, as shown in Supplementary Table 13.

### Oxford Nanopore Technology Exhibits Multiple G4 Motifs in *VERNALIZATION1*

Given the importance of G-quadruplex structures in the regulation of gene transcription (Cagirici and Sen, 2020), we used Quadruplex forming G-Rich Sequences (QGRS) mapper for detecting G4 motifs within the three homoeologous *VRN1* genes and their promoters on sense strand. Four G4 motifs within *VRN-A1* were found in all tested cultivars, one G4 motif was located within the promoter and three G4 motifs were located within the first intron. Sanger sequencing showed that the G4 motif located within the promoter had one SNP (SNP3) carrying the combination of two alleles (C/G) in five cultivars (GT5), Ivanka, Renesansa, NS 66/92, Premio, and BCD 1302/83 (Supplementary Table 10 and Supplementary Figure 7). For *VRN-B1*, one G4 motif within the promoter and two G4 motifs within the first intron were identified in all cultivars. However, in four cultivars, INTRO 615, NS 22/92, Cappelle Desprez and Highbury, the G4 motif located 5,099 bp downstream of the start codon was missing due to the presence of a large deletion in their first intron. For *VRN-D1*, two motifs in the promoter and one in the first intron were detected. With the exception of the SNP3 in the G4 motif in the *VRN-A1* promoter, ONT and Sanger sequencing did not show any polymorphism in other G4 motif sequences across the investigated cultivars.

### Polymorphisms in *VERNALIZATION1* Homoeologs Are Associated With Multiple Agronomic Traits Across Spring and Winter Wheat

The SVs, single nucleotide variation and CNV detected within the three homoelogous *VRN1* sequences divide the 190 cultivars into different sequence type groups, genotype groups and haplotype groups. Firstly, we performed an association study between the SVs and the phenotypes for 20 agronomic traits. In this analysis, seven cultivars carrying the known SV spring alleles (*Vrn-A1a*, *Vrn-B1a*) were grouped together (SV1 group). Eight cultivars carrying only the novel SV allele *Vrn-B1f* were grouped together (SV2 group). Three out of eight within group SV2 have been described to show a spring type growth habit and five out of eight have been described to show a winter type growth habit. The remaining 175 cultivars without any SVs were gathered in another group (intact allele group, 173 winter and 2 spring types). The association study showed that both SV groups (SV1 and SV2) are significantly associated with an increase of powdery mildew disease, with decreased grain yield, decreased biomass, decreased protein yield, decreased nitrogen use efficiency (NUE), decreased RUE, decreased RIE, with increased crude protein, increased plant height and early heading date. Furthermore, group SV1 (*Vrn-A1a*, *Vrn-B1a*) showed a significant association with, decreased falling number, decreased TKW, and decreased harvest index. Group SV2 (*Vrn-B1f* allele) showed a significant association with decreased GCD and decreased kernels per m^2^ (Supplementary Figure 11). For *vrn-D1r* allele, resulting from 17 bp deletion in intron 1 of the *VRN-D1*, no significant association with any of the studied traits was found.

### Polymorphism in *VERNALIZATION1* Homoeologs Are Associated With Multiple Agronomic Traits in Winter Wheat

**FIGURE 5:**
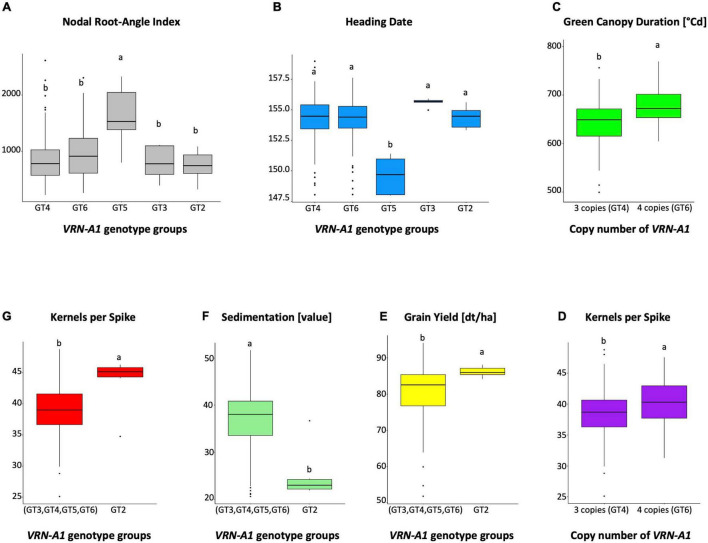
Boxplots showing pairwise comparisons between genotype groups **(A,B,E,F,G)** and copy numbers **(C,D)** for *VRN-A1* gene and different traits. Columns labeled with different letters represent significant difference at *P* ≤ 0.05.

Next, we analyzed only cultivars which have been described to show the winter type growth habit to find polymorphisms which exist in adapted winter wheat cultivars and are correlated with agronomic traits of interest that are not obviously imparted by a strong difference in plant phenology. For this reason, we excluded twelve cultivars from analysis which either have been described by CYMMT (Genetic Resources Information System for Wheat and Triticale, 2022) or others to display a spring-type growth habit, or cultivars which showed a known spring allele (*Vrn-A1a*, *Vrn-B1a*) harboring a promoter insertion or an intron deletion. Five winter-type cultivars which carried a novel *Vrn-B1f* allele harboring the 838 bp duplication were included in the analysis. The remaining 178 cultivars with a clear winter-type growth habit showed a similarly high phenotypic variation for all traits compared to the complete set of 190 putative spring and winter type cultivars. For *VRN-B1*, the haplotype Hap4 carrying a novel SNP at position 8615 was not significantly associated with any trait. For *VRN-A1* we found that genotype group GT2, which is very different from other genotype groups (GT3, GT4, GT5, and GT6) with 30 polymorphisms, was associated with significantly increased grain yield, increased kernels per spike and increased kernels per m^2^, and with decreased sedimentation value and crude protein content (which are generally negatively correlated to grain yield; Figures 5E–G). The *VRN-A1* sequence type group “Weebill” consists of four genotype groups (Table 3). The genotype group GT3 which carries a homozygous C allele at SNP45 within exon 4 was negatively correlated with grain yield, harvest index, TKW, and positively correlated with sedimentation value, plant height, crude protein content and heading date. The two heterozygous SNPs, SNP3 (CG), and SNP22 (GA), within the promoter and intron 1 region of GT5, were significantly associated with early heading date, increased nodal root-angle index NRI (Figures 5A,B), susceptibility to powdery mildew disease, decreased grain yield, decreased biomass, decreased protein yield, decreased NUE, and decreased RIE. With the exception of GT3 group, the three genotype groups GT4, GT5, and GT6 differ from each other by four heterozygous SNPs. This suggests together with the KASP analysis above and previous reports that gene CNV is in involved in association between these genotype groups and agronomic traits. An association study of four haplotypes (four copies) of GT6 versus two haplotypes (three copies) of GT4 for *VRN-A1* (Table 4) with the traits showed that the presence of four copies was significantly associated with an increased green canopy duration, increased kernels per spike (Figures 5C,D), increased harvest index, increased RIE and with decreased crude protein and plant height.

## Discussion

We showed that long-read sequencing of multiplexed long-PCR amplicons is feasible for simultaneous genetic analysis of the full length of homoeologous *VRN1* genes from a hexaploid wheat population. This approach enables cost-effective and simple population-wide analysis of candidate genes based on the use of only two indexed PCR primers to amplify the full-length sequence of multiple homoeologous genes in one PCR reaction. In contrast, in the past, a set of 25 primers has been applied to sequence only one *VRN-A1* gene (13,600 bp) using Sanger sequencing technology (Ivaničová et al., 2016). One limitation of the applied ONT multiplexing approach that should be addressed in future research is the presence of the high number of reads lacking an identifiable barcode (unclassified reads) due to high error rate of ONT data and classification limitations of current demultiplexing tools (Wick et al., 2018). Another limitation of ONT multiplexing is the production of a high number of chimeric reads leading to cross-barcode assignment errors. Previous studies reported that chimeric reads are major source of erroneous sequence assignments in ONT data when long PCR amplicons are sequenced. Formation of chimeric reads can originate from PCR amplification, but has been mainly reported to originate from the ONT sequencing process (Ashelford et al., 2006; White et al., 2017; Xu et al., 2018). However, our experiment demonstrated that the amount of data generated through ONT from two flow cells was sufficient for population-wide analysis for *VRN1* genes of 192 wheat cultivars. In the past, the low per base-accuracy of ONT data has made reliable SNP calling questionable with tools developed for short read variants (Ameur et al., 2019). However, quality of ONT reads is constantly increasing and new long-read variant callers are being developed (Møller et al., 2020; Ahsan et al., 2021). By comparison with Sanger sequencing data we showed here that reliable SNP calling using the long-read variant caller Medaka is possible for genes sequenced with a coverage above 11x. Similarly, Vollrath et al. (2021) have shown that SNP calling based on the long-read variant caller Clair (Luo et al., 2020) and an average genome-wide coverage rate above 23x allowed calling of high-confidence SNPs from ONT data in a polyploid crop genome.

Using long read sequencing allowed us to reconstruct full-length *VRN1* genes and analyze the structure of *VRN1* homoeologous genes. We observed that the two known dominant spring alleles *Vrn-A1a*, *Vrn-B1a* (Yan et al., 2004; Fu et al., 2005) are present either together or alone in seven cultivars originating from the United States, United Kingdom, Mexico, France, Serbia described in the literature mostly as spring type, but also with some conflicting classifications. All 192 cultivars including these seven survived winter under German growing conditions in six locations and two growing seasons (Voss-Fels et al., 2019b). Winter hardiness or freezing tolerance was reported to be the most important physiological trait next to vernalization requirement used to describe a winter wheat in a survey of international wheat breeders (Crofts, 1989). A major frost tolerance locus is known to co-map with the *VRN1* locus on chromosome 5A (Zhao et al., 2013; Zhu et al., 2014; Babben et al., 2018). However, all of these seven diverse cultivars classified by genetics as spring types due to the presence of the dominant *Vrn-A1a*, *Vrn-B1a* alleles, showed very poor agronomic performance under German winter growing conditions and are not adapted winter wheat types. Similar findings have been reported before (Kamran et al., 2013, 2014; Kollers et al., 2013). Thus, not all tested cultivars surviving winter in Germany can be classified genetically as winter types.

We found nine cultivars harboring a recently described allele, *Vrn-B1f* (Strejčková et al., 2021) in four spring and five winter type cultivars released in different geographic regions (Germany, Chile, Mexico, and Australia). Like the dominant *Vrn-A1a* and *Vrn-B1a* alleles, this allele is associated with a reduction in many agronomic traits (e.g., grain yield, kernels per m^2^, protein yield, NUE). Strejčková et al. (2021) detected the *Vrn-B1f* allele in three spring wheat genotypes. In our study, one of the cultivars which carry this allele showed a combination with the dominant spring allele *Vrn-A1a*, while the other eight only carried the *Vrn-B1f* allele. Strejčková et al. (2021) suggested that the new *Vrn-B1f* allele is a dominant spring allele. However, in the three genotypes where they detected the *Vrn-B1f* alleles, it was combined with the dominant strong *Vrn-A1a* spring allele. From eight cultivars which do not show a combination with *Vrn-A1a* in our study, only 3 behave like spring types and 5 like winter types, suggesting that this allele might be not sufficient to create a spring type growth habit. In the past, using short read sequencing, this allele escaped detection because an 838 bp duplication can only be assayed with long-reads. We also discovered a novel allele we termed *Vrn-D1x* within the first intron of *VRN-D1* in two Mexican cultivars Mex. 3 and Mex. 17 bb. However, because this novel allele was detected in only two cultivars and in combination with the spring allele *Vrn-A1a*, the effect of the novel allele on agronomic traits could not be estimated. We developed a KASP assay and PCR assays which can be used to detect individuals carrying the *Vrn-D1x* and *Vrn-B1f* in marker-assisted breeding.

Chen et al. (2011) reported that cultivars carrying the dominant spring *Vrn-A1a* polymorphism also carry the C allele at SNP51 in exon 7 in *VRN-A1*, while those carrying the recessive winter *vrn-A1* allele carry the mutated T allele at this position. Our detailed analysis of the molecular polymorphism for *VRN-A1* is not completely consistent with this result, as we found that 6 out of 11 cultivars which carry the C allele at this position were harboring the recessive *vrn-A1* allele, while the other five cultivars were carrying the dominant *Vrn-A1a* allele. Similar findings were reported by Muterko and Salina (2018) who showed that most, but not all of hexaploid wheat genotypes carrying the recessive *vrn-A1* allele have a T allele in exon 7, while almost all known dominant *Vrn-A1* alleles are carrying the C allele in exon 7. We conclude that this polymorphism in exon 7 is not a good predictor for spring/winter type growth habit as reported by Chen et al. (2011).

As expected, due to major effects of phenology, the known dominant SV alleles distinguishing spring from winter ecotypes were found to be correlated with 13 of the 20 tested traits. Cultivars carrying SVs within *VRN1* genes showed association with many traits such as lower grain yield, biomass and harvest index compared to those with intact genes. It also has been described before that *VRN1* polymorphism is associated with vernalization response, grain yield, spikelet number per spike, spike development, thousand grain weight, stem elongation, heading date, NRI and other developmental traits (Kamran et al., 2013, 2014; Zheng et al., 2013; Zhang et al., 2014; Voss-Fels et al., 2018; Li et al., 2019; Chumanova et al., 2020; Dreisigacker et al., 2021; Sheoran et al., 2022). Analysis of SV spring alleles together with novel SV alleles revealed correlations with seven traits which have not been evaluated before, e.g., powdery mildew infection, kernels per m^2^, falling number, RUE, NUE, RIE and GCD. This can be expected as *VRN1* is known to be a key regulator of reproductive growth and floral initiation and known to be involved in major developmental differences, which in turn can have direct or indirect effects on many agronomic traits. Likely most of the traits listed above found to be correlated with *VRN1* polymorphism are due to indirect and not causal effects. E.g., acceleration in development within the *VRN1*-triggered transition from vegetative to reproductive phase can result in an increase of infection to diseases at the time point of infection. This also has been reported for photoperiod insensitive *Ppd-D1a* mutants showing decreased resistance against Fusarium infection in wheat (Kollers et al., 2013). The vast majority of previous studies focused on analyzing the effect of dominant *Vrn1* alleles on agronomic traits. One likely reason for this was that the known large structural polymorphisms (deletions/insertions) within dominant alleles were easy to screen in germplasm using traditional PCR analysis. Here we describe new SNPs and new deletion/insertion polymorphisms within winter-type cultivars which escaped detection so far and show that they are associated with numerous agronomic traits. SNP variation and CNV within winter types was found to be correlated with 16 of 20 of the tested traits. In addition, to the traits found to be correlated for the complete cultivar panel, sedimentation value, nodal root-angle index NRI and kernels per spike was also found to be correlated when using 178 winter types for analysis. Cultivars classified as genotype group GT2 Haplotyp 2 (Robigus, Claire, Xanthippe, Capone, SUR99820, Sponsor) exhibiting a *VRN-A1* gene with very high SNP divergence compared to other winter types show a low sedimentation value, low crude protein content and high grain yield, kernels per spike and kernels per m^2^.

Previous studies showed that winter wheat was heading and flowering late and that vernalization requirement was increased in biparental crosses carrying three instead of two or one copy of *VRN-A1* (Díaz et al., 2012; Guedira et al., 2016). We did not find correlation between copy number and heading in a diverse winter wheat panel. This is in agreement with the study of Würschum et al. (2015) and Dixon et al. (2019) who did not find an effect of *VRN-A1* CNV on flowering time. Díaz et al. (2012); Dixon et al. (2019), and Muterko and Salina (2021) showed an effect of CNV on vernalization requirement. We found strong correlations with six other agronomic traits. The presence of four copies (4 haplotypes, GT6) showed positive effects, while three copies (2 haplotypes, GT4) showed negative effects on green canopy duration, kernels per spike, harvest index, and radiation interception efficiency. The negative effect might be due to the presence of the additional copies in mutated forms (T allele). Similarly, Dixon et al. (2019) hypothesized that varieties with different composition of C and T alleles in exon 4 of *VRN-A1* are involved in vernalization acting under different temperatures. It was suggested that the variation in expression between the C and T allele in exon 4 has additional functions in the regulation of *VRN-A1* expression. Deng et al. (2015) identified over 500 genomic regions as potential *VRN1*-binding targets in the wheat genome. This suggest that a mutated non-functional copy of *VRN1* might impact not just the vernalization response, but also many other pathways involved in developmental processes affecting many different traits. Only 6 of the 178 tested winter type cultivars carried one copy of the non-mutated *VRN-A1* allele (C allele at exon 4 and exon 7). These are the same cultivars mentioned above to carry Haplotype 2. Some of them are high-yielding successful cultivars such as Robigus and Claire. One trait with high interest in increasing yield potential of winter wheat is kernels per spike (Li et al., 2019; Voss-Fels et al., 2019a) which showed an average of around 12 percent increase between cultivars from Haplotype 2 and other winter type cultivars. Targeting the Haplotype 2 and CNV by marker-assisted breeding can be a promising approach to introduce these rare positive alleles into winter wheat germplasm. For this we have developed simple breeder-friendly KASP assays for SNP and CNV detection (VRN5A-SNP5K, VRN5A-SNP18K, VRN5A-SNP45K, and VRN5A-SNP51K). Cultivars classified as genotype group GT5 mostly originating from Eastern Europe show earlier flowering time and a higher NRI value. NRI indicates a higher fraction of nodal roots at deeper root angles corresponding to narrower, deeper roots. These cultivars show a strong contrast to all other winter type cultivars (two-fold difference in average NRI value). The correlation with NRI supports the hypothesis of Voss-Fels et al. (2018) that novel molecular variants of *VRN1*, distinct from the major winter-spring polymorphism, are responsible for modulation of root development in winter wheat germplasm. Genotype group 5 shows two polymorphisms, one of them located in the G4 motif in promoter and another one in intron 1. The G4 motif has been reported to play an important role in gene expression regulation and post-transcriptional regulation (Du et al., 2008; Bugaut and Balasubramanian, 2012). Breeding for wheat resilient to climate change has been suggest to target root architecture and flowering time (Sheehan and Bentley, 2020; Ober et al., 2021; Rambla et al., 2022). Earlier flowering winter wheat cultivars with a deeper root system allowing greater access to soil moisture during water deficit could help to avoid drought stress. We developed a KASP marker (VRN5A-SNP22K) which can be applied in marker-assisted breeding to introduce these traits from these five identified cultivars with extreme root architecture.

## Data Availability Statement

The datasets presented in this study can be found in online repositories. The names of the repository/repositories and accession number(s) can be found below: NCBI (accession: PRJNA839568).

## Author Contributions

CO and RS conceived the idea. MM, HC, and CO developed the methodology, performed data curation, and analyzed the data. RS sourced the funding. BW, AS, KV-F, and HZ produced and provided phenotype data. MM and CO drafted the manuscript. All authors revised the manuscript, contributed to the article, and approved the submitted version.

## Conflict of Interest

The authors declare that the research was conducted in the absence of any commercial or financial relationships that could be construed as a potential conflict of interest.

## Publisher’s Note

All claims expressed in this article are solely those of the authors and do not necessarily represent those of their affiliated organizations, or those of the publisher, the editors and the reviewers. Any product that may be evaluated in this article, or claim that may be made by its manufacturer, is not guaranteed or endorsed by the publisher.

## Supplementary Material

Supplementary Figure 1 Flowchart for three pipelines applied on ONT sequencing data to generate full-length *VRN1* sequences using a combination of different software packages (Okonechnikov et al., 2012; Katoh and Standley, 2013; Koren et al., 2017; Sedlazeck et al., 2018). *De novo* assembly (Canu) command used in the A_denovo and B_align_denovo pipelines for most barcodes was “*canu -p output_filename_prefix -d output_directory_name genomeSize* = *80k*^A_denovo^* or 25k*^B_align_denovo^* -nanopore-raw inputfile.fastq correctedErrorRate* = *0.105 minReadLength* = *2000 minOverlapLength* = *1000 corOutCoverage* = *6000 corMhapSensitivity* = *normal corMinCoverage* = *0 “batOptions* = *-dg 3 -db 3 -dr 1 -ca 500 -cp 50.”* For four cultivars showing a large deletion of 6,851 bp within the first intron of *VRN-B1* gene, the filtering step “Read > 11 kbp” was skipped from C_align pipeline.

Supplementary Figure 2 Characteristics of data generated by multiplex ONT sequencing of *VRN1* homoeologous copies from 192 wheat cultivars. **(A)** Distribution of yield (11.2 Gb) of raw sequence data for different types of reads. **(B)** Mean coverage of *VRN1* genes with only uniquely mapping reads, and distribution of reads larger than 10,500 bp on three *VRN1* genes. Black bars represent standard deviation.

Supplementary Figure 3 Multiple alignment of *VRN-B1* sequences obtained by ONT sequencing using B_align_denovo pipeline shows a 838 bp duplication in the first intron in nine cultivars. The sequence of LongReach Lancer and Robigus cultivars are published by the wheat 10 + genome project.

Supplementary Figure 4 Multiple alignment of *VRN-D1* sequences obtained by Sanger sequencing shows a 163 bp insertion within the first intron. Bold letters represent the insertion.

Supplementary Figure 5 Multiple alignment of *VRN-D1* sequences obtained by Sanger sequencing shows a 17 bp deletion in the first intron in 15 cultivars.

Supplementary Figure 6 Multiple alignment of *VRN-A1* sequences obtained by Sanger sequencing shows a heterozygous SNP, SNP22 at position 4757 bp in the first intron in five cultivars carrying genotype group GT5.

Supplementary Figure 7 Multiple alignment of *VRN-A1* sequences obtained by Sanger sequencing shows a heterozygous SNP, SNP3 at position 381 bp in the promoter in five cultivars carrying the genotype group GT5.

Supplementary Figure 8 Multiple alignment of *VRN-A1* sequences obtained by Sanger sequencing shows a heterozygous SNP, SNP18 at position 4,138 bp in the first intron in four cultivars carrying the genotype group GT6.

Supplementary Figure 9 Multiple alignment of *VRN-A1* sequences obtained by Sanger sequencing shows different polymorphisms detected in intron 2, exon 4, and intron 4. SNP45 highlighted in yellow color is heterozygous at position 11,109 bp in exon 4.

Supplementary Figure 10 Multiple alignment of *VRN-B1* sequences obtained by Sanger sequencing shows a SNP at position 8,615 bp in the first intron (11 cultivars carrying A allele at this SNP locus assigned to haplotype Hap4).

Supplementary Figure 11 Boxplots showing pairwise comparisons between groups with different structural variation for *VRN1* and different traits. Columns labeled with different letters represent significant difference at *P* ≤ 0.05.

Supplementary Table 1 Barcode information and detailed information about origin, country of registration, breeder and seed quality as reported by Lichthardt et al. (2020) and Voss-Fels et al. (2019b) and phenotype data as reported by Voss-Fels et al. (2019b) and Voss-Fels et al. (2018).

Supplementary Table 2 Primer sequences.

Supplementary Table 3 KASP primer sequences for *VRN1.*

Supplementary Table 4 Characteristics of data generated by multiplex ONT sequencing for three *VRN1* homoeologous loci from 192 wheat cultivars.

Supplemenarty Table 5 Best blast hits of the full-length *VRN-A1* sequences generated applying three different pipelines with 32 reference genomes using ncbi-blast-2.6.0+.

Supplementary Table 6 Best blast hits of the full-length *VRN-B1* sequences generated by applying three different pipelines with 29 reference genomes using ncbi-blast-2.6.0+.

Supplementary Table 7 Best blast hits of the full-length *VRN-D1* sequences generated by applying three different pipelines with 22 reference genomes using ncbi-blast-2.6.0+.

Supplementary Table 8 Percentage identity, number of poymorhisom and structural varaitions within the *VRN1* sequences of five wheat references obtained from publicly available wheat reference genome sequence data (IPK Crop Analysis Tools Suite, 2021), and NCBI database (Accession number AY747601.1).

Supplementary Table 9 Grouping of 192 wheat cultivars based on top BLASTn hits of full-length *VRN1* sequences with around 30 reference genomes reveal 9 groups of A, B, and D homeolog sequence combinations most similar (>99.9%) to only five reference genomes.

Supplementary Table 10 G-quadruplex (G4 motif) identified in promoter and the first intron of the three homoeologus *VRN1* genes in 192 wheat cultivars using QGRS Mapper software.

Supplementary Table 11 Comparison of copy number estimates for *VRN-A1* for 190 cultivars based on different methods.

Supplementary Table 12 Cultivars with structural variations at three *VRN1* loci detected in this study.

Supplementary Table 13 Genotypes and haplotypes of the homoeologus *VRN1* loci for 190 bread wheat cultivars.
