# Supplementary material for: Long-Amplicon Single-Molecule Sequencing Reveals Novel, Trait-Associated Variants of VERNALIZATION1 Homoeologs in Hexaploid Wheat
Source: Front Plant Sci. 2022 Jul 15;13:942461. doi: 10.3389/fpls.2022.942461 (PMC9676936; doi:10.3389/fpls.2022.942461)
Supplement: Supplementary Figure 1 — Flowchart for three pipelines applied on ONT sequencing data to generate full-length VRN1 sequences using a combination of different software packages (Okonechnikov et al., 2012; Katoh and Standley, 2013; Koren et al., 2017; Sedlazeck et al., 2018). De novo assembly (Canu) command used in the A_denovo and B_align_denovo pipelines for most barcodes was “canu -p output_filename_prefix -d output_directory_name genomeSize = 80kA_denovo or 25kB_align_denovo -nanopore-raw inputfile.fastq correctedErrorRate = 0.105 minReadLength = 2000 minOverlapLength = 1000 corOutCoverage = 6000 corMhapSensitivity = normal corMinCoverage = 0 “batOptions = -dg 3 -db 3 -dr 1 -ca 500 -cp 50.” For four cultivars showing a large deletion of 6,851 bp within the first intron of VRN-B1 gene, the filtering step “Read > 11 kbp” was skipped from C_align pipeline. [file Data_Sheet_1.pdf]

De novo assembly and consensus sequence generation from Oxford Nanopore Technologies (ONT) data

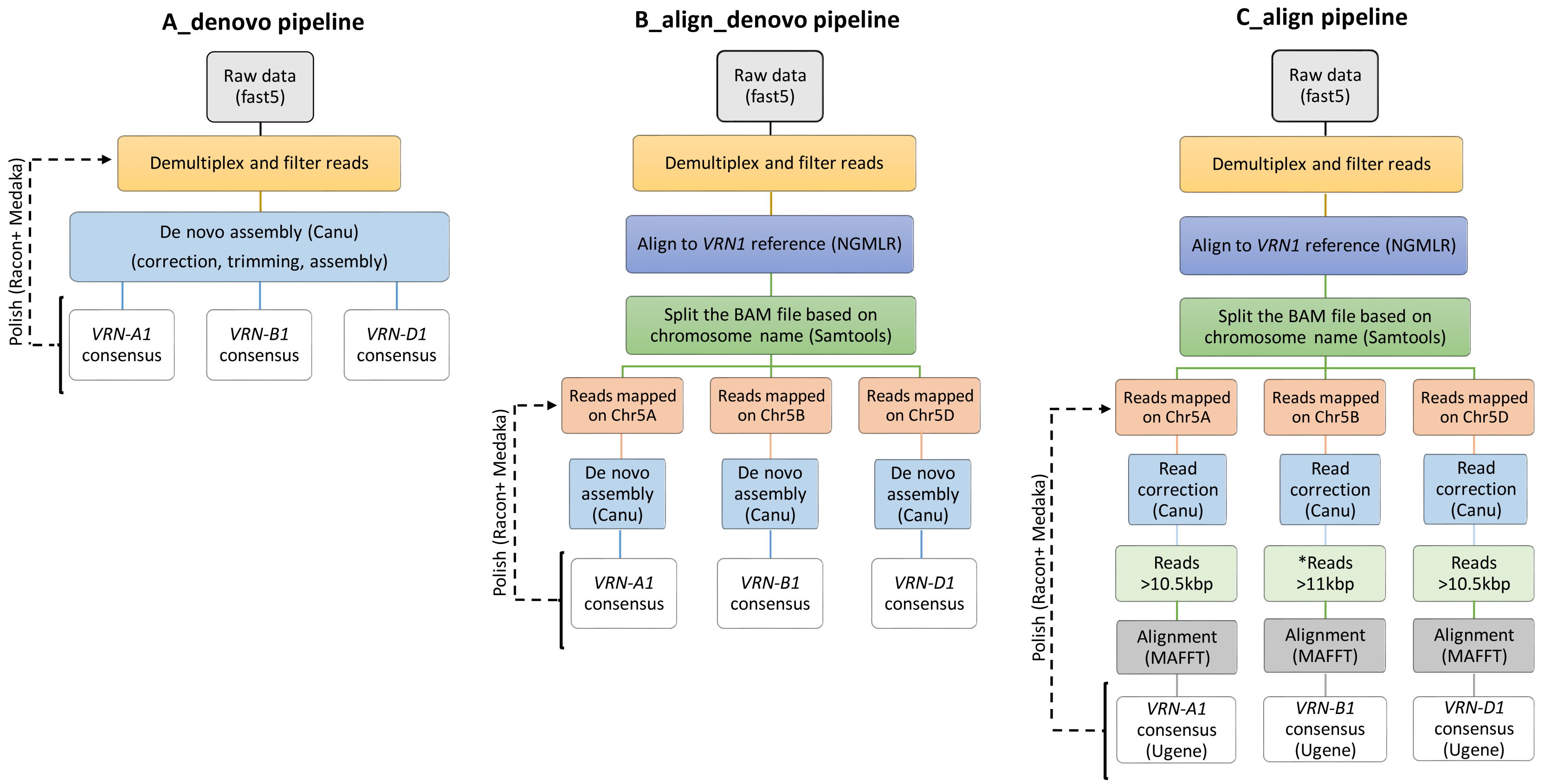

Supplementary Figure 1 Flowchart for three pipelines applied on ONT sequencing data to generate full-length *VRN1* sequences using a combination of different software packages (Koren et al., 2017; Katoh and Standley, 2013; Okonechnikov et al., 2012; Sedlazeck et al., 2018). *De-novo* assembly (Canu) command used in the A_denovo and B_align_denovo pipelines for most barcodes was "*canu -p output_filename_prefix -d output_directory_name genomeSize=80k*$^{A\_denovo}$ *or 25k*$^{B\_align\_denovo}$ *-nanopore-raw inputfile.fastq correctedErrorRate=0.105 minReadLength=2000 minOverlapLength=1000 corOutCoverage=6000 corMhapSensitivity=normal corMinCoverage=0 "batOptions=-dg 3 -db 3 -dr 1 -ca 500 -cp 50"*. For four cultivars showing a large deletion of 6851 bp within the first intron of *VRN-B1* gene, the filtering step "Read >11kbp" was skipped from C_align pipeline.

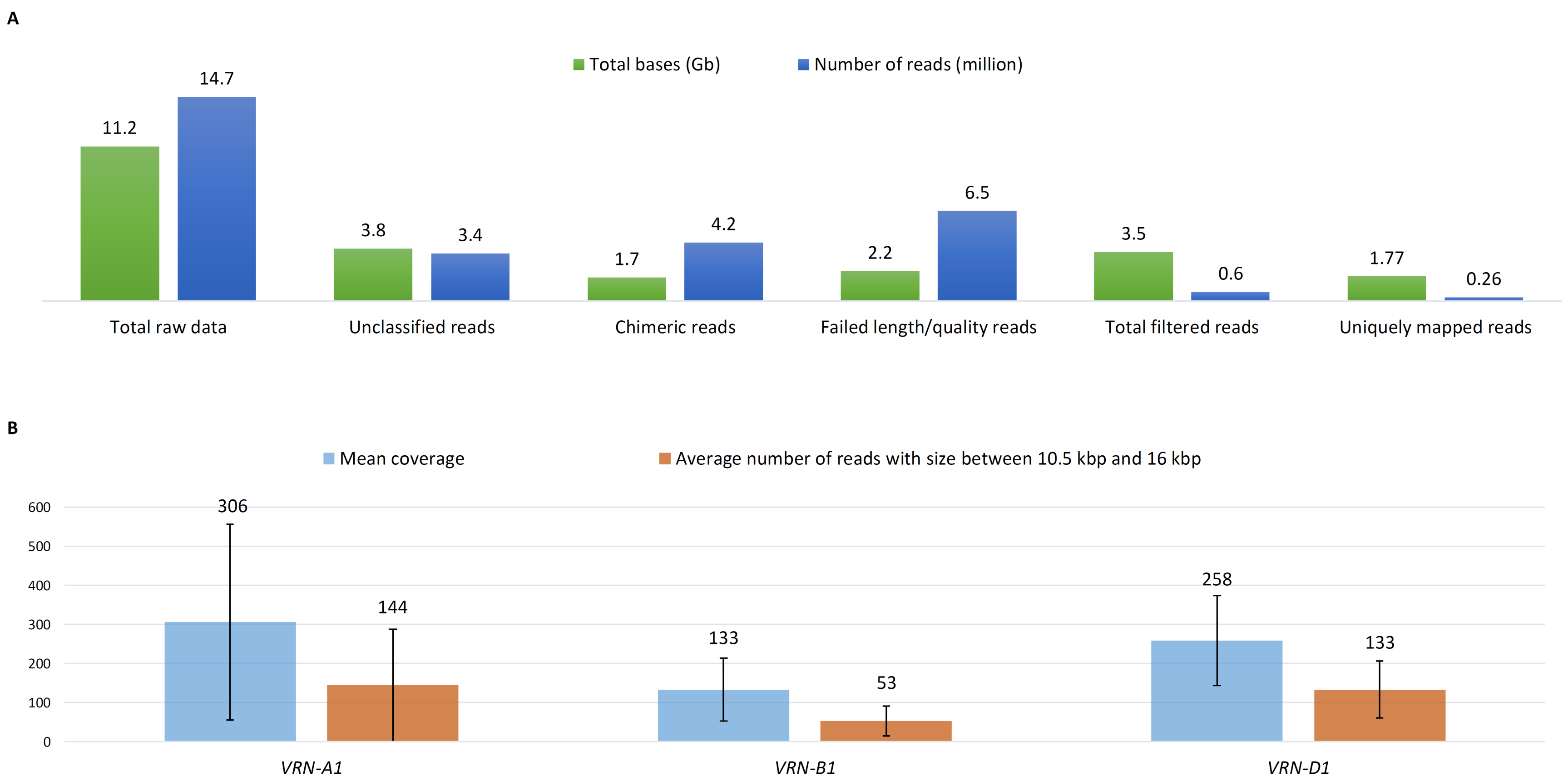

Supplementary Figure 2 Characteristics of data generated by multiplex ONT sequencing of *VRN1* homoeologous copies from 192 wheat cultivars. A) Distribution of yield (11.2 Gb) of raw sequence data for different types of reads. B) Mean coverage of *VRN1* genes with only uniquely mapping reads, and distribution of reads larger than 10500 bp on three *VRN1* genes. Black bars represent standard deviation.

Longreach lancer	ATTCACATGTGACTAGTGTTCTGCCCCTGTTTAGGAATGAAATCGATGCTTCTGAACCAG
Robigus EIV1.1	ATTCACATGTGACTAGTGTTCTGCCCCTGTTTAGGAATGAAATCGATGCTTCTGAACCAG
Lambriego	ATTCACATGTGACTAGTGTTCTGCCCCTGTTTAGGAATGAAATCGATGCTTCTGAACCAG
Naturastar	ATTCACATGTGACTAGTGTTCTGCCCCTGTTTAGGAATGAAATCGATGCTTCTGAACCAG
Phoenix	ATTCACATGTGACTAGTGTTCTGCCCCTGTTTAGGAATGAAATCGATGCTTCTGAACCAG
Topfit	ATTCACATGTGACTAGTGTTCTGCCCCTGTTTAGGAATGAAATCGATGCTTCTGAACCAG
Joss	ATTCACATGTGACTAGTGTTCTGCCCCTGTTTAGGAATGAAATCGATGCTTCTGAACCAG
Cajeme	ATTCACATGTGACTAGTGTTCTGCCCCTGTTTAGGAATGAAATCGATGCTTCTGAACCAG
Tambor	ATTCACATGTGACTAGTGTTCTGCCCCTGTTTAGGAATGAAATCGATGCTTCTGAACCAG
Triple	ATTCACATGTGACTAGTGTTCTGCCCCTGTTTAGGAATGAAATCGATGCTTCTGAACCAG
Mex.3	ATTCACATGTGACTAGTGTTCTGCCCCTGTTTAGGAATGAAATCGATGCTTCTGAACCAG
	**
Longreach lancer	TTCAATGTGTTTGTTGGATATTTTTTGGAGAAGAACATGTGACCCAATGACAGGTGGGTC
Robigus EIV1.1	TTCAATGTGTTTGTTGGATATTTTTTGGAGAAGAACATGTGACCCAATGACAGGTGGGTC
Lambriego	TTCAATGTGTTTGTTGGATATTTTTTGGAGAAGAACATGTGACCCAATGACAGGTGGGTC
Naturastar	TTCAATGTGTTTGTTGGATATTTTTTGGAGAAGAACATGTGACCCAATGACAGGTGGGTC
Phoenix	TTCAATGTGTTTGTTGGATATTTTTTGGAGAAGAACATGTGACCCAATGACAGGTGGGTC
Topfit	TTCAATGTGTTTGTTGGATATTTTTTGGAGAAGAACATGTGACCCAATGACAGGTGGGTC
Joss	TTCAATGTGTTTGTTGGATATTTTTTGGAGAAGAACATGTGACCCAATGACAGGTGGGTC
Cajeme	TTCAATGTGTTTGTTGGATATTTTTTGGAGAAGAACATGTGACCCAATGACAGGTGGGTC
Tambor	TTCAATGTGTTTGTTGGATATTTTTTGGAGAAGAACATGTGACCCAATGACAGGTGGGTC
Triple	TTCAATGTGTTTGTTGGATATTTTTTGGAGAAGAACATGTGACCCAATGACAGGTGGGTC
Mex.3	TTCAATGTGTTTGTTGGATATTTTTTGGAGAAGAACATGTGACCCAATGACAGGTGGGTC
	**
Longreach lancer	CTACTCGTAGGGCACGACTCTCTGTTTGCGCTTTGGATAGGCATCCTCCATATCCATATG
Robigus EIV1.1	CTACTCGTAGGGCACGACTCTCTGTTTGCGCTTTGGATAGGCATCCTCCATATCCATATG
Lambriego	CTACTCGTAGGGCACGACTCTCTGTTTGCGCTTTGGATAGGCATCCTCCATATCCATATG
Naturastar	CTACTCGTAGGGCACGACTCTCTGTTTGCGCTTTGGATAGGCATCCTCCATATCCATATG
Phoenix	CTACTCGTAGGGCACGACTCTCTGTTTGCGCTTTGGATAGGCATCCTCCATATCCATATG
Topfit	CTACTCGTAGGGCACGACTCTCTGTTTGCGCTTTGGATAGGCATCCTCCATATCCATATG
Joss	CTACTCGTAGGGCACGACTCTCTGTTTGCGCTTTGGATAGGCATCCTCCATATCCATATG
Cajeme	CTACTCGTAGGGCACGACTCTCTGTTTGCGCTTTGGATAGGCATCCTCCATATCCATATG
Tambor	CTACTCGTAGGGCACGACTCTCTGTTTGCGCTTTGGATAGGCATCCTCCATATCCATATG
Triple	CTACTCGTAGGGCACGACTCTCTGTTTGCGCTTTGGATAGGCATCCTCCATATCCATATG
Mex.3	CTACTCGTAGGGCACGACTCTCTGTTTGCGCTTTGGATAGGCATCCTCCATATCCATATG
	**
Longreach lancer	GAGCGCATCAACGTGGTGACTCTGCTAGAGTTGCTCTTACACACCTCGATATGCAACTTT
Robigus EIV1.1	GAGCGCATCAACGTGGTGACTCTGCTAGAGTTGCTCTTACACACCTCGATATGCAACTTT
Lambriego	GAGCGCATCAACGTGGTGACTCTGCTAGAGTTGCTCTTACACACCTCGATATGCAACTTT
Naturastar	GAGCGCATCAACGTGGTGACTCTGCTAGAGTTGCTCTTACACACCTCGATATGCAACTTT
Phoenix	GAGCGCATCAACGTGGTGACTCTGCTAGAGTTGCTCTTACACACCTCGATATGCAACTTT
Topfit	GAGCGCATCAACGTGGTGACTCTGCTAGAGTTGCTCTTACACACCTCGATATGCAACTTT
Joss	GAGCGCATCAACGTGGTGACTCTGCTAGAGTTGCTCTTACACACCTCGATATGCAACTTT
Cajeme	GAGCGCATCAACGTGGTGACTCTGCTAGAGTTGCTCTTACACACCTCGATATGCAACTTT
Tambor	GAGCGCATCAACGTGGTGACTCTGCTAGAGTTGCTCTTACACACCTCGATATGCAACTTT
Triple	GAGCGCATCAACGTGGTGACTCTGCTAGAGTTGCTCTTACACACCTCGATATGCAACTTT
Mex.3	GAGCGCATCAACGTGGTGACTCTGCTAGAGTTGCTCTTACACACCTCGATATGCAACTTT
	**
Longreach lancer	AGCTCACCATGCTTGATATACGTTTAGCCATCCAATGATTGAACAATTGGTGTTGGTAGA
Robigus EIV1.1	AGCTCACCATGCTTGATATACGTTTAGCCATCCAATGATTGAACAATTGGTGTTGGTAGA
Lambriego	AGCTCACCATGCTTGATATACGTTTAGCCATCCAATGATTGAACAATTGGTGTTGGTAGA
Naturastar	AGCTCACCATGCTTGATATACGTTTAGCCATCCAATGATTGAACAATTGGTGTTGGTAGA
Phoenix	AGCTCACCATGCTTGATATACGTTTAGCCATCCAATGATTGAACAATTGGTGTTGGTAGA
Topfit	AGCTCACCATGCTTGATATACGTTTAGCCATCCAATGATTGAACAATTGGTGTTGGTAGA
Joss	AGCTCACCATGCTTGATATACGTTTAGCCATCCAATGATTGAACAATTGGTGTTGGTAGA
Cajeme	AGCTCACCATGCTTGATATACGTTTAGCCATCCAATGATTGAACAATTGGTGTTGGTAGA
Tambor	AGCTCACCATGCTTGATATACGTTTAGCCATCCAATGATTGAACAATTGGTGTTGGTAGA
Triple	AGCTCACCATGCTTGATATACGTTTAGCCATCCAATGATTGAACAATTGGTGTTGGTAGA
Mex.3	AGCTCACCATGCTTGATATACGTTTAGCCATCCAATGATTGAACAATTGGTGTTGGTAGA
	**
Longreach lancer	AGCAGAGTCCATGCCAAACCACTATGTTA**AATGCCTGACAAACGGGTCTTTATCTAGTTC**
Robigus EIV1.1	AGCAGAGTCCATGCCAAACCACTATGTTA-------------------------------
Lambriego	AGCAGAGTCCATGCCAAACCACTATGTTA**AATGCCTGACAAACGGGTCTTTATCTAGTTC**
Naturastar	AGCAGAGTCCATGCCAAACCACTATGTTA**AATGCCTGACAAACGGGTCTTTATCTAGTTC**
Phoenix	AGCAGAGTCCATGCCAAACCACTATGTTA**AATGCCTGACAAACGGGTCTTTATCTAGTTC**
Topfit	AGCAGAGTCCATGCCAAACCACTATGTTA**AATGCCTGACAAACGGGTCTTTATCTAGTTC**
Joss	AGCAGAGTCCATGCCAAACCACTATGTTA**AATGCCTGACAAACGGGTCTTTATCTAGTTC**
Cajeme	AGCAGAGTCCATGCCAAACCACTATGTTA**AATGCCTGACAAACGGGTCTTTATCTAGTTC**

```
Tambor             AGCAGAGTCCATGCCAAACCACTATGTTAAATGCCTGACAAACGGGTCTTTATCTAGTTC
Triple             AGCAGAGTCCATGCCAAACCACTATGTTAAATGCCTGACAAACGGGTCTTTATCTAGTTC
Mex.3              AGCAGAGTCCATGCCAAACCACTATGTTAAATGCCTGACAAACGGGTCTTTATCTAGTTC
                   *****************************

Longreach lancer   CTTTTTGAGATGCACCTTAGTGTGTTATTTTCTAGGGTTGCCTCACCATTGGGATCCCTT
Robigus EIV1.1     ------------------------------------------------------------
Lambriego          CTTTTTGAGATGCACCTTAGTGTGTTATTTTCTAGGGTTGCCTCACCATTGGGATCCCTT
Naturastar         CTTTTTGAGATGCACCTTAGTGTGTTATTTTCTAGGGTTGCCTCACCATTGGGATCCCTT
Phoenix            CTTTTTGAGATGCACCTTAGTGTGTTATTTTCTAGGGTTGCCTCACCATTGGGATCCCTT
Topfit             CTTTTTGAGATGCACCTTAGTGTGTTATTTTCTAGGGTTGCCTCACCATTGGGATCCCTT
Joss               CTTTTTGAGATGCACCTTAGTGTGTTATTTTCTAGGGTTGCCTCACCATTGGGATCCCTT
Cajeme             CTTTTTGAGATGCACCTTAGTGTGTTATTTTCTAGGGTTGCCTCACCATTGGGATCCCTT
Tambor             CTTTTTGAGATGCACCTTAGTGTGTTATTTTCTAGGGTTGCCTCACCATTGGGATCCCTT
Triple             CTTTTTGAGATGCACCTTAGTGTGTTATTTTCTAGGGTTGCCTCACCATTGGGATCCCTT
Mex.3              CTTTTTGAGATGCACCTTAGTGTGTTATTTTCTAGGGTTGCCTCACCATTGGGATCCCTT

Longreach lancer   GGGTCGGACCCTTCCCCGACCCTGCGCAAGCGGGAGCTACATGCACCGGGCTGCCC-TTT
Robigus EIV1.1     ------------------------------------------------------------
Lambriego          GGGTCGGACCCTTCCCCGACCCTGCGCAAGCGGGAGCTACATGCACCGGGCTGCCC-TTT
Naturastar         GGGTCGGACCCTTCCCCGACCCTGCGCAAGCGGGAGCTACATGCACCGGGCTGCCC-TTT
Phoenix            GGGTCGGACCCTTCCCCGACCCTGCGCAAGCGGGAGCTACATGCACCGGGCTGCCC-TTT
Topfit             GGGTCGGACCCTTCCCCGACCCTGCGCAAGCGGGAGCTACATGCACCGGGCTGCCC-TTT
Joss               GGGTCGGACCCTTCCCCGACCCTGCGCAAGCGGGAGCTACATGCACCGGGCTGCCCTTTT
Cajeme             GGGTCGGACCCTTCCCCGACCCTGCGCAAGCGGGAGCTACATGCACCGGGCTGCCC--TT
Tambor             GGGTCGGACCCTTCCCCGACCCTGCGCAAGCGGGAGCTACATGCACCGGGCTGCCC---T
Triple             GGGTCGGACCCTTCCCCGACCCTGCGCAAGCGGGAGCTACATGCACCGGGCTGCCC-TTT
Mex.3              GGGTCGGACCCTTCCCCGACCCTGCGCAAGCGGGAGCTACATGCACCGGGCTGCCCTTTT

Longreach lancer   TTTTTTCACATGCCTTCAATCCACCA-CCCCCTTTGTTGTTCATGGCGCATCCGGAGGAG
Robigus EIV1.1     ------------------------------------------------------------
Lambriego          TTTTTTCACATGCCTTCAATCCACCA-CCCCCTTTGTTGTTCATGGCGCATCCGGAGGAG
Naturastar         TTTTTTCACATGCCTTCAATCCACCA-CCCCCTTTGTTGTTCATGGCGCATCCGGAGGAG
Phoenix            TTTTTTCACATGCCTTCAATCCACCA-CCCCCTTTGTTGTTCATGGCGCATCCGGAGGAG
Topfit             TTTTTTCACATGCCTTCAATCCACCA-CCCCCTTTGTTGTTCATGGCGCATCCGGAGGAG
Joss               TTTTTTCACATGCCTTCAATCCACCA-CCCCCTTTGTTGTTCATGGCGCATCCGGAGGAG
Cajeme             TTTTTTCACATGCCTTCAATCCACCA-CCCCCTTTGTTGTTCATGGCGCATCCGGAGGAG
Tambor             TTTTTTCACATGCCTTCAATCCACCA-CCCCCTTTGTTGTTCATGGCGCATCCGGAGGAG
Triple             TTTTTTCACATGCCTTCAATCCACCA-CCCCCTTTGTTGTTCATGGCGCATCCGGAGGAG
Mex.3              TTTTTTCACATGCCTTCAATCCACCACCCCCCTTTGTTGTTCATGGCGCATCCGGAGGAG

Longreach lancer   CTCTCTCTCTCATCTTCAATTTTTGGCATGAGGTGGAGTAGCCCGCGGATCCTTTT-CCC
Robigus EIV1.1     ------------------------------------------------------------
Lambriego          CTCTCTCTCTCATCTTCAATTTTTGGCATGAGGTGGAGTAGCCCGCGGATCCTTTT-CCC
Naturastar         CTCTCTCTCTCATCTTCAATTTTTGGCATGAGGTGGAGTAGCCCGCGGATCCTTTT--CC
Phoenix            CTCTCTCTCTCATCTTCAATTTTTGGCATGAGGTGGAGTAGCCCGCGGATCCTTTTCCCC
Topfit             CTCTCTCTCTCATCTTCAATTTTTGGCATGAGGTGGAGTAGCCCGCGGATCCTTTT-CCC
Joss               CTCTCTCTCTCATCTTCAATTTTTGGCATGAGGTGGAGTAGCCCGCGGATCCTTTT--CC
Cajeme             --CTCTCTCTCATCTTCAATTTTTGGCATGAGGTGGAGTAGCCCGCGGATCCTTTT--CC
Tambor             CTCTCTCTCTCATCTTCAATTTTTGGCATGAGGTGGAGTAGCCCGCGGATCCTTTT--CC
Triple             CTCTCTCTCTCATCTTCAATTTTTGGCATGAGGTGGAGTAGCCCGCGGATCCTTTT-CCC
Mex.3              CTCTCTCTCTCATCTTCAATTTTTGGCATGAGGTGGAGTAGCCCGCGGATCCTTTT--CC

Longreach lancer   CCCTTTAGGGACCAGATGGACCTAGTCCTGGAGGAGGCCGCGCAGATGGAAGCGCAAATG
Robigus EIV1.1     ------------------------------------------------------------
Lambriego          CCCTTTAGGGACCAGATGGACCTAGTCCTGGAGGAGGCCGCGCAGATGGAAGCGCAAATG
Naturastar         CCCTTTAGGGACCAGATGGACCTAGTCCTGGAGGAGGCCGCGCAGATGGAAGCGCAAATG
Phoenix            CCCTTTAGGGACCAGATGGACCTAGTCCTGGAGGAGGCCGCGCAGATGGAAGCGCAAATG
Topfit             CCCTTTAGGGACCAGATGGACCTAGTCCTGGAGGAGGCCGCGCAGATGGAAGCGCAAATG
Joss               CCCTTTAGGGACCAGATGGACCTAGTCCTGGAGGAGGCCGCGCAGATGGAAGCGCAAATG
Cajeme             CCCTTTAGGGACCAGATGGACCTAGTCCTGGAGGAGGCCGCGCAGATGGAAGCGCAAATG
Tambor             CCCTTTAGGGACCAGATGGACCTAGTCCTGGAGGAGGCCGCGCAGATGGAAGCGCAAATG
Triple             CCCTTTAGGGACCAGATGGACCTAGTCCTGGAGGAGGCCGCGCAGATGGAAGCGCAAATG
Mex.3              CCCTTTAGGGACCAGATGGACCTAGTCCTGGAGGAGGCCGCGCAGATGGAAGCGCAAATG

Longreach lancer   GAGCAGCAGATTTGGGCACTAGCACACTCCCGCCGCCGCGGATGAATGGCCGGCCTGGGA
Robigus EIV1.1     ------------------------------------------------------------
Lambriego          GAGCAGCAGATTTGGGCACTAGCACACTCCCGCCGCCGCGGATGAATGGCCGGCCTGGGA
```

```
Naturastar       GAGCAGCAGATTTGGGCACTAGCACACTCCCGCCGCCGCGGATGAATGGCCGGCCTGGGA
Phoenix          GAGCAGCAGATTTGGGCACTAGCACACTCCCGCCGCCGCGGATGAATGGCCGGCCTGGGA
Topfit           GAGCAGCAGATTTGGGCACTAGCACACTCCCGCCGCCGCGGATGAATGGCCGGCCTGGGA
Joss             GAGCAGCAGATTTGGGCACTAGCACACTCCCGCCGCCGCGGATGAATGGCCGGCCTGGGA
Cajeme           GAGCAGCAGATTTGGGCACTAGCACACTCCCGCCGCCGCGGATGAATGGCCGGCCTGGGA
Tambor           GAGCAGCAGATTTGGGCACTAGCACACTCCCGCCGCCGCGGATGAATGGCCGGCCTGGGA
Triple           GAGCAGCAGATTTGGGCACTAGCACACTCCCGCCGCCGCGGATGAATGGCCGGCCTGGGA
Mex.3            GAGCAGCAGATTTGGGCACTAGCACACTCCCGCCGCCGCGGATGAATGGCCGGCCTGGGA

Longreach lancer CAACGTCGCTGAGGCGCATTTCCAAGTCATAGTGGCCAACATTGTTGCATTTTACGTAGG
Robigus EIV1.1   ------------------------------------------------------------
Lambriego        CAACGTCGCTGAGGCGCATTTCCAAGTCATAGTGGCCAACATTGTTGCATTTTACGTAGG
Naturastar       CAACGTCGCTGAGGCGCATTTCCAAGTCATAGTGGCCAACATTGTTGCATTTTACGTAGG
Phoenix          CAACGTCGCTGAGGCGCATTTCCAAGTCATAGTGGCCAACATTGTTGCATTTTACGTAGG
Topfit           CAACGTCGCTGAGGCGCATTTCCAAGTCATAGTGGCCAACATTGTTGCATTTTACGTAGG
Joss             CAACGTCGCTGAGGCGCATTTCCAAGTCATAGTGGCCAACATTGTTGCATTTTACGTAGG
Cajeme           CAACGTCGCTGAGGCGCATTTCCAAGTCATAGTGGCCAACATTGTTGCATTTTACGTAGG
Tambor           CAACGTCGCTGAGGCGCATTTCCAAGTCATAGTGGCCAACATTGTTGCATTTTACGTAGG
Triple           CAACGTCGCTGAGGCGCATTTCCAAGTCATAGTGGCCAACATTGTTGCATTTTACGTAGG
Mex.3            CAACGTCGCTGAGGCGCATTTCCAAGTCATAGTGGCCAACATTGTTGCATTTTACGTAGG

Longreach lancer TGGTGGCAGTGGTCGTGGCCGCAACTAGTTTAACTAATTATTAGGTGTTCTATGAATGTA
Robigus EIV1.1   ------------------------------------------------------------
Lambriego        TGGTGGCAGTGGTCGTGGCCGCAACTAGTTTAACTAATTATTAGGTGTTCTATGAATGTA
Naturastar       TGGTGGCAGTGGTCGTGGCCGCAACTAGTTTAACTAATTATTAGGTGTTCTATGAATGTA
Phoenix          TGGTGGCAGTGGTCGTGGCCGCAACTAGTTTAACTAATTATTAGGTGTTCTATGAATGTA
Topfit           TGGTGGCAGTGGTCGTGGCCGCAACTAGTTTAACTAATTATTAGGTGTTCTATGAATGTA
Joss             TGGTGGCAGTGGTCGTGGCCGCAACTAGTTTAACTAATTATTAGGTGTTCTATGAATGTA
Cajeme           TGGTGGCAGTGGTCGTGGCCGCAACTAGTTTAACTAATTATTAGGTGTTCTATGAATGTA
Tambor           TGGTGGCAGTGGTCGTGGCCGCAACTAGTTTAACTAATTATTAGGTGTTCTATGAATGTA
Triple           TGGTGGCAGTGGTCGTGGCCGCAACTAGTTTAACTAATTATTAGGTGTTCTATGAATGTA
Mex.3            TGGTGGCAGTGGTCGTGGCCGCAACTAGTTTAACTAATTATTAGGTGTTCTATGAATGTA

Longreach lancer ATTCACATGTGACTAGTGTTCCGCCCCTGTTTAGGAATGAAATCGATGCTTCTGAACCAG
Robigus EIV1.1   ------------------------------------------------------------
Lambriego        ATTCACATGTGACTAGTGTTCCGCCCCTGTTTAGGAATGAAATCGATGCTTCTGAACCAG
Naturastar       ATTCACATGTGACTAGTGTTCCGCCCCTGTTTAGGAATGAAATCGATGCTTCTGAACCAG
Phoenix          ATTCACATGTGACTAGTGTTCCGCCCCTGTTTAGGAATGAAATCGATGCTTCTGAACCAG
Topfit           ATTCACATGTGACTAGTGTTCCGCCCCTGTTTAGGAATGAAATCGATGCTTCTGAACCAG
Joss             ATTCACATGTGACTAGTGTTCCGCCCCTGTTTAGGAATGAAATCGATGCTTCTGAACCAG
Cajeme           ATTCACATGTGACTAGTGTTCCGCCCCTGTTTAGGAATGAAATCGATGCTTCTGAACCAG
Tambor           ATTCACATGTGACTAGTGTTCCGCCCCTGTTTAGGAATGAAATCGATGCTTCTGAACCAG
Triple           ATTCACATGTGACTAGTGTTCCGCCCCTGTTTAGGAATGAAATCGATGCTTCTGAACCAG
Mex.3            ATTCACATGTGACTAGTGTTCCGCCCCTGTTTAGGAATGAAATCGATGCTTCTGAACCAG

Longreach lancer TTCAATGTGTTTGTTGGATATTTTTTGGAGAAGAACATGTGACCCAATGACAGGTGGGTC
Robigus EIV1.1   ------------------------------------------------------------
Lambriego        TTCAATGTGTTTGTTGGATATTTTTTGGAGAAGAACATGTGACCCAATGACAGGTGGGTC
Naturastar       TTCAATGTGTTTGTTGGATATTTTTTGGAGAAGAACATGTGACCCAATGACAGGTGGGTC
Phoenix          TTCAATGTGTTTGTTGGATATTTTTTGGAGAAGAACATGTGACCCAATGACAGGTGGGTC
Topfit           TTCAATGTGTTTGTTGGATATTTTTTGGAGAAGAACATGTGACCCAATGACAGGTGGGTC
Joss             TTCAATGTGTTTGTTGGATATTTTTTGGAGAAGAACATGTGACCCAATGACAGGTGGGTC
Cajeme           TTCAATGTGTTTGTTGGATATTTTTTGGAGAAGAACATGTGACCCAATGACAGGTGGGTC
Tambor           TTCAATGTGTTTGTTGGATATTTTTTGGAGAAGAACATGTGACCCAATGACAGGTGGGTC
Triple           TTCAATGTGTTTGTTGGATATTTTTTGGAGAAGAACATGTGACCCAATGACAGGTGGGTC
Mex.3            TTCAATGTGTTTGTTGGATATTTTTTGGAGAAGAACATGTGACCCAATGACAGGTGGGTC

Longreach lancer CTACTCGTAGGGCACGACTCTCTGTTTGCGCTTTGGATAGGCATCCTCCATATCCATATG
Robigus EIV1.1   ------------------------------------------------------------
Lambriego        CTACTCGTAGGGCACGACTCTCTGTTTGCGCTTTGGATAGGCATCCTCCATATCCATATG
Naturastar       CTACTCGTAGGGCACGACTCTCTGTTTGCGCTTTGGATAGGCATCCTCCATATCCATATG
Phoenix          CTACTCGTAGGGCACGACTCTCTGTTTGCGCTTTGGATAGGCATCCTCCATATCCATATG
Topfit           CTACTCGTAGGGCACGACTCTCTGTTTGCGCTTTGGATAGGCATCCTCCATATCCATATG
Joss             CTACTCGTAGGGCACGACTCTCTGTTTGCGCTTTGGATAGGCATCCTCCATATCCATATG
Cajeme           CTACTCGTAGGGCACGACTCTCTGTTTGCGCTTTGGATAGGCATCCTCCATATCCATATG
Tambor           CTACTCGTAGGGCACGACTCTCTGTTTGCGCTTTGGATAGGCATCCTCCATATCCATATG
Triple           CTACTCGTAGGGCACGACTCTCTGTTTGCGCTTTGGATAGGCATCCTCCATATCCATATG
Mex.3            CTACTCGTAGGGCACGACTCTCTGTTTGCGCTTTGGATAGGCATCCTCCATATCCATATG
```

```
Longreach lancer GAGCGCATCAACGTGGTGACTCTGCTAGAGTTGCTCTTACACACCTCGATATGCAACTTT
Robigus EIV1.1   ------------------------------------------------------------
Lambriego        GAGCGCATCAACGTGGTGACTCTGCTAGAGTTGCTCTTACACACCTCGATATGCAACTTT
Naturastar       GAGCGCATCAACGTGGTGACTCTGCTAGAGTTGCTCTTACACACCTCGATATGCAACTTT
Phoenix          GAGCGCATCAACGTGGTGACTCTGCTAGAGTTGCTCTTACACACCTCGATATGCAACTTT
Topfit           GAGCGCATCAACGTGGTGACTCTGCTAGAGTTGCTCTTACACACCTCGATATGCAACTTT
Joss             GAGCGCATCAACGTGGTGACTCTGCTAGAGTTGCTCTTACACACCTCGATATGCAACTTT
Cajeme           GAGCGCATCAACGTGGTGACTCTGCTAGAGTTGCTCTTACACACCTCGATATGCAACTTT
Tambor           GAGCGCATCAACGTGGTGACTCTGCTAGAGTTGCTCTTACACACCTCGATATGCAACTTT
Triple           GAGCGCATCAACGTGGTGACTCTGCTAGAGTTGCTCTTACACACCTCGATATGCAACTTT
Mex.3            GAGCGCATCAACGTGGTGACTCTGCTAGAGTTGCTCTTACACACCTCGATATGCAACTTT

Longreach lancer AGCTCACCATGCTTGATATACGTTTAGCCATCCAATGATTGAACAATTGGTGTTGGTAGA
Robigus_EIV1.1   ------------------------------------------------------------
Lambriego        AGCTCACCATGCTTGATATACGTTTAGCCATCCAATGATTGAACAATTGGTGTTGGTAGA
Naturastar       AGCTCACCATGCTTGATATACGTTTAGCCATCCAATGATTGAACAATTGGTGTTGGTAGA
Phoenix          AGCTCACCATGCTTGATATACGTTTAGCCATCCAATGATTGAACAATTGGTGTTGGTAGA
Topfit           AGCTCACCATGCTTGATATACGTTTAGCCATCCAATGATTGAACAATTGGTGTTGGTAGA
Joss             AGCTCACCATGCTTGATATACGTTTAGCCATCCAATGATTGAACAATTGGTGTTGGTAGA
Cajeme           AGCTCACCATGCTTGATATACGTTTAGCCATCCAATGATTGAACAATTGGTGTTGGTAGA
Tambor           AGCTCACCATGCTTGATATACGTTTAGCCATCCAATGATTGAACAATTGGTGTTGGTAGA
Triple           AGCTCACCATGCTTGATATACGTTTAGCCATCCAATGATTGAACAATTGGTGTTGGTAGA
Mex.3            AGCTCACCATGCTTGATATACGTTTAGCCATCCAATGATTGAACAATTGGTGTTGGTAGA

Longreach lancer AGCAGAGTCCATGCCAAACCACTATGTTCAATGCCTGACAAACGGGTCTTTATCTAGTTC
Robigus EIV1.1   -----------------------------AATGCCTGACAAACGGGTCTTTATCTAGTTC
Lambriego        AGCAGAGTCCATGCCAAACCACTATGTTCAATGCCTGACAAACGGGTCTTTATCTAGTTC
Naturastar       AGCAGAGTCCATGCCAAACCACTATGTTCAATGCCTGACAAACGGGTCTTTATCTAGTTC
Phoenix          AGCAGAGTCCATGCCAAACCACTATGTTCAATGCCTGACAAACGGGTCTTTATCTAGTTC
Topfit           AGCAGAGTCCATGCCAAACCACTATGTTCAATGCCTGACAAACGGGTCTTTATCTAGTTC
Joss             AGCAGAGTCCATGCCAAACCACTATGTTCAATGCCTGACAAACGGGTCTTTATCTAGTTC
Cajeme           AGCAGAGTCCATGCCAAACCACTATGTTCAATGCCTGACAAACGGGTCTTTATCTAGTTC
Tambor           AGCAGAGTCCATGCCAAACCACTATGTTCAATGCCTGACAAACGGGTCTTTATCTAGTTC
Triple           AGCAGAGTCCATGCCAAACCACTATGTTCAATGCCTGACAAACGGGTCTTTATCTAGTTC
Mex.3            AGCAGAGTCCATGCCAAACCACTATGTTCAATGCCTGACAAACGGGTCTTTATCTAGTTC
                                              *******************************

Longreach lancer CTTTTTGAGATGCACCTTAGTGTGTTATTTTCTAGGGTTGCCTCACCATTGGGATCCCTT
Robigus EIV1.1   CTTTTTGAGATGCACCTTAGTGTGTTATTTTCTAGGGTTGCCTCACCATTGGGATCCCTT
Lambriego        CTTTTTGAGATGCACCTTAGTGTGTTATTTTCTAGGGTTGCCTCACCATTGGGATCCCTT
Naturastar       CTTTTTGAGATGCACCTTAGTGTGTTATTTTCTAGGGTTGCCTCACCATTGGGATCCCTT
Phoenix          CTTTTTGAGATGCACCTTAGTGTGTTATTTTCTAGGGTTGCCTCACCATTGGGATCCCTT
Topfit           CTTTTTGAGATGCACCTTAGTGTGTTATTTTCTAGGGTTGCCTCACCATTGGGATCCCTT
Joss             C-TTTTGAGATGCACCTTAGTGTGTTATTTTCTAGGGTTGCCTCACCATTGGGATCCCTT
Cajeme           CTTTTTGAGATGCACCTTAGTGTGTTATTTTCTAGGGTTGCCTCACCATTGGGATCCCTT
Tambor           CTTTTTGAGATGCACCTTAGTGTGTTATTTTCTAGGGTTGCCTCACCATTGGGATCCCTT
Triple           CTTTTTGAGATGCACCTTAGTGTGTTATTTTCTAGGGTTGCCTCACCATTGGGATCCCTT
Mex.3            CTTTTTGAGATGCACCTTAGTGTGTTATTTTCTAGGGTTGCCTCACCATTGGGATCCCTT
                 * **********************************************************

Longreach lancer GGGATCACCTTACTATGTGCATAATTTCTTTTCACATGGGCATTTATCTGCTTTGGCATT
Robigus EIV1.1   GGGATCACCTTACTATGTGCATAATTTCTTTTCACATGGGCATTTATCTGCTTTGGCATT
Lambriego        GGGATCACCTTACTATGTGCATAATTTCTTTTCACATGGGCATTTATCTGCTTTGGCATT
Naturastar       GGGATCACCTTACTATGTGCATAATTTCTTTTCACATGGGCATTTATCTGCTTTGGCATT
Phoenix          GGGATCACCTTACTATGTGCATAATTTCTTTTCACATGGGCATTTATCTGCTTTGGCATT
Topfit           GGGATCACCTTACTATGTGCATAATTTCTTTTCACATGGGCATTTATCTGCTTTGGCATT
Joss             GGGATCACCTTACTATGTGCATAATTTCTTTTCACATGGGCATTTATCTGCTTTGGCATT
Cajeme           GGGATCACCTTACTATGTGCATAATTTCTTTTCACATGGGCATTTATCTGCTTTGGCATT
Tambor           GGGATCACCTTACTATGTGCATAATTTCTTTTCACATGGGCATTTATCTGCTTTGGCATT
Triple           GGGATCACCTTACTATGTGCATAATTTCTTTTCACATGGGCATTTATCTGCTTTGGCATT
Mex.3            GGGATCACCTTACTATGTGCATAATTTCTTTTCACATGGGCATTTATCTGCTTTGGCATT
                 ************************************************************
```

Supplementary Figure 3 Multiple alignment of *VRN-B1* sequences obtained by ONT sequencing using B_align_denovo pipeline shows a 838 bp duplication in the first intron in nine cultivars. The sequence of LongReach Lancer and Robigus cultivars are published by the wheat 10+ genome project.

```
Mex. 17 bb Vrn-D1x   AACCAACCGCCAACGTGGGGTGGAAGTTTTAGGCCCTGCTTGGAATGGGTGTAAGTTTTT
Mex. 3 Vrn-D1x       AACCAACCGCCAACGTGGGGTGGAAGTTTTAGGCCCTGCTTGGAATGGGTGTAAGTTTTT
Hermann vrn-D1       AACCAACCGCCAACGTGGGGTGGAAGT---------------------------------
                     ***************************

Mex. 17 bb Vrn-D1x   ACACCTGTATTTATCTTACAGGTGTAAATTACTGGTCGCCACTTGATTCCAGCCTGAAAT
Mex. 3 Vrn-D1x       ACACCTGTATTTATCTTACAGGTGTAAATTACTGGTCGCCACTTGATTCCAGCCTGAAAT
Hermann vrn-D1       ------------------------------------------------------------


Mex. 17 bb Vrn-D1x   TAAAACGACGGAAAGAGGTCCGTATCCTAAACCGGCCAAAAACGAGCTGAAACGCTGATC
Mex. 3 Vrn-D1x       TAAAACGACGGAAAGAGGTCCGTATCCTAAACCGGCCAAAAACGAGCTGAAACGCTGATC
Hermann vrn-D1       ------------------------------------------------------------


Mex. 17 bb Vrn-D1x   CAAGCAGGGCTTTAAGCTTCCTTCTGCCGCCTCCCGTCGGATTTGAAGCAGCTCGGCCTC
Mex. 3 Vrn-D1x       CAAGCAGGGCTTTAAGCTTCCTTCTGCCGCCTCCCGTCGGATTTGAAGCAGCTCGGCCTC
Hermann vrn-D1       ----------TTTAAGCTTCCTTCTGCCGCCTCCCGTCGGATTTGAAGCAGCTCGGCCTC
                               **************************************************
```

Supplementary Figure 4 Multiple alignment of *VRN-D1* sequences obtained by Sanger sequencing shows a 163 bp insertion within the first intron. Bold letters represent the insertion

```
Benni multifloret vrn-D1   TTTTGAATATTCAAACTGACATGTATTAAAGACGTATACATGTGTCTTTATATCACATGT
Konsul vrn-D1r             TTTTGAATATTCAAACTGACATGTATTAAAGACGTAT-----------------ACATGT
Robigus vrn-D1r            TTTTGAATATTCAAACTGACATGTATTAAAGACGTAT-----------------ACATGT
Muskat vrn-D1r             TTTTGAATATTCAAACTGACATGTATTAAAGACGTAT-----------------ACATGT
Arlequin vrn-D1r           TTTTGAATATTCAAACTGACATGTATTAAAGACGTAT-----------------ACATGT
Disponent vrn-D1r          TTTTGAATATTCAAACTGACATGTATTAAAGACGTAT-----------------ACATGT
Einstein vrn-D1r           TTTTGAATATTCAAACTGACATGTATTAAAGACGTAT-----------------ACATGT
Capone vrn-D1r             TTTTGAATATTCAAACTGACATGTATTAAAGACGTAT-----------------ACATGT
Enorm vrn-D1r              TTTTGAATATTCAAACTGACATGTATTAAAGACGTAT-----------------ACATGT
JB Asano vrn-D1r           TTTTGAATATTCAAACTGACATGTATTAAAGACGTAT-----------------ACATGT
Sperber vrn-D1r            TTTTGAATATTCAAACTGACATGTATTAAAGACGTAT-----------------ACATGT
Diplomat vrn-D1r           TTTTGAATATTCAAACTGACATGTATTAAAGACGTAT-----------------ACATGT
Benno vrn-D1r              TTTTGAATATTCAAACTGACATGTATTAAAGACGTAT-----------------ACATGT
Gourmet vrn-D1r            TTTTGAATATTCAAACTGACATGTATTAAAGACGTAT-----------------ACATGT
Nimbus vrn-D1r             TTTTGAATATTCAAACTGACATGTATTAAAGACGTAT-----------------ACATGT
Helios vrn-D1r             TTTTGAATATTCAAACTGACATGTATTAAAGACGTAT-----------------ACATGT
                           *************************************                 ******

Benni multifloret vrn-D1   GTGCTTTTCCTGCTTAAGGTAGTAGGACTATCTCTTTTTTGAGAAACACCGATTACAACG
Konsul vrn-D1r             GTGCTTTTCCTGCTTAAGGTAGTAGGACTATCTCTTTTTTGAGAAACACCGATTACAACG
Robigus vrn-D1r            GTGCTTTTCCTGCTTAAGGTAGTAGGACTATCTCTTTTTTGAGAAACACCGATTACAACG
Muskat vrn-D1r             GTGCTTTTCCTGCTTAAGGTAGTAGGACTATCTCTTTTTTGAGAAACACCGATTACAACG
Arlequin vrn-D1r           GTGCTTTTCCTGCTTAAGGTAGTAGGACTATCTCTTTTTTGAGAAACACCGATTACAACG
Disponent vrn-D1r          GTGCTTTTCCTGCTTAAGGTAGTAGGACTATCTCTTTTTTGAGAAACACCGATTACAACG
Einstein vrn-D1r           GTGCTTTTCCTGCTTAAGGTAGTAGGACTATCTCTTTTTTGAGAAACACCGATTACAACG
Capone vrn-D1r             GTGCTTTTCCTGCTTAAGGTAGTAGGACTATCTCTTTTTTGAGAAACACCGATTACAACG
Enorm vrn-D1r              GTGCTTTTCCTGCTTAAGGTAGTAGGACTATCTCTTTTTTGAGAAACACCGATTACAACG
JB Asano vrn-D1r           GTGCTTTTCCTGCTTAAGGTAGTAGGACTATCTCTTTTTTGAGAAACACCGATTACAACG
Sperber vrn-D1r            GTGCTTTTCCTGCTTAAGGTAGTAGGACTATCTCTTTTTTGAGAAACACCGATTACAACG
Diplomat vrn-D1r           GTGCTTTTCCTGCTTAAGGTAGTAGGACTATCTCTTTTTTGAGAAACACCGATTACAACG
Benno vrn-D1r              GTGCTTTTCCTGCTTAAGGTAGTAGGACTATCTCTTTTTTGAGAAACACCGATTACAACG
Gourmet vrn-D1r            GTGCTTTTCCTGCTTAAGGTAGTAGGACTATCTCTTTTTTGAGAAACACCGATTACAACG
Nimbus vrn-D1r             GTGCTTTTCCTGCTTAAGGTAGTAGGACTATCTCTTTTTTGAGAAACACCGATTACAACG
Helios vrn-D1r             GTGCTTTTCCTGCTTAAGGTAGTAGGACTATCTCTTTTTTGAGAAACACCGATTACAACG
                           ************************************************************
```

Supplementary Figure 5 Multiple alignment of *VRN-D1* sequences obtained by Sanger sequencing shows a 17 bp deletion in the first intron in 15 cultivars.

```
Renesansa        TGGTTATCATCTAGTACTTTCTGTACCACGTTTATCTTACAAACGCRAGGGTATTGTGGT
Premio           TGGTTATCATCTAGTACTTTCTGTACCACGTTTATCTTACAAACGCRAGGGTATTGTGGT
BCD 1302/83      TGGTTATCATCTAGTACTTTCTGTACCACGTTTATCTTACAAACGCRAGGGTATTGTGGT
NS 66/92         TGGTTATCATCTAGTACTTTCTGTACCACGTTTATCTTACAAACGCRAGGGTATTGTGGT
Ivanka           TGGTTATCATCTAGTACTTTCTGTACCACGTTTATCTTACAAACGCRAGGGTATTGTGGT
Apache           TGGTTATCATCTAGTACTTTCTGTACCACGTTTATCTTACAAACGCGAGGGTATTGTGGT
Isengrain        TGGTTATCATCTAGTACTTTCTGTACCACGTTTATCTTACAAACGCGAGGGTATTGTGGT
                 ********************************************** *************

Renesansa        GGAGAGCCATGTATTACAACTTTGATAATCAAAATTCAAAATTCAAGTCTCTGAAGATCC
Premio           GGAGAGCCATGTATTACAACTTTGATAATCAAAATTCAAAATTCAAGTCTCTGAAGATCC
BCD 1302/83      GGAGAGCCATGTATTACAACTTTGATAATCAAAATTCAAAATTCAAGTCTCTGAAGATCC
NS 66/92         GGAGAGCCATGTATTACAACTTTGATAATCAAAATTCAAAATTCAAGTCTCTGAAGATCC
Ivanka           GGAGAGCCATGTATTACAACTTTGATAATCAAAATTCAAAATTCAAGTCTCTGAAGATCC
Apache           GGAGAGCCATGTATTACAACTTTGATAATCAAAATTCAAAATTCAAGTCTCTGAAGATCC
Isengrain        GGAGAGCCATGTATTACAACTTTGATAATCAAAATTCAAAATTCAAGTCTCTGAAGATCC
                 ************************************************************
```

Supplementary Figure 6 Multiple alignment of *VRN-A1* sequences obtained by Sanger sequencing shows a heterozygous SNP, SNP22 at position 4757 bp in the first intron in five cultivars carrying genotype group GT5.

```
Sponsor          AATATTATGAAAGGAAAAATTCTGCTCGTTTTTTTGCTCTGTGGTGTGTGTTTGTGGCGA
Renesansa        AATATTATGAAAGGAAAAATTCTGCTCGTTTTTTTGCTCTGTGGTGTGTGTTTGTGGCGA
Premio           AATATTATGAAAGGAAAAATTCTGCTCGTTTTTTTGCTCTGTGGTGTGTGTTTGTGGCGA
BCD 1302/83      AATATTATGAAAGGAAAAATTCTGCTCGTTTTTTTGCTCTGTGGTGTGTGTTTGTGGCGA
NS 66/92         AATATTATGAAAGGAAAAATTCTGCTCGTTTTTTTGCTCTGTGGTGTGTGTTTGTGGCGA
Ivanka           AATATTATGAAAGGAAAAATTCTGCTCGTTTTTTTGCTCTGTGGTGTGTGTTTGTGGCGA
Kormoran         AATATTATGAAAGGAAAAATTCTGCTCGTTTTTTTGCTCTGTGGTGTGTGTTTGTGGCGA
                 ************************************************************

Sponsor          GAGAAAATGATTTGGGGAAAGCAAAATCCGGAGATTCGCACGTACGATCGTTCGACACGT
Renesansa        GAGAAAATGATTTGGGGAAAGCAAAATCCGGAGATTCGCACGTACGATCGTTCGACACGT
Premio           GAGAAAATGATTTGGGGAAAGCAAAATCCGGAGATTCGCACGTACGATCGTTCGACACGT
BCD 1302/83      GAGAAAATGATTTGGGGAAAGCAAAATCCGGAGATTCGCACGTACGATCGTTCGACACGT
NS 66/92         GAGAAAATGATTTGGGGAAAGCAAAATCCGGAGATTCGCACGTACGATCGTTCGACACGT
Ivanka           GAGAAAATGATTTGGGGAAAGCAAAATCCGGAGATTCGCACGTACGATCGTTCGACACGT
Kormoran         GAGAAAATGATTTGGGGAAAGCAAAATCCGGAGATTCGCACGTACGATCGTTCGACACGT
                 ************************************************************

Sponsor          CGACGCCCGGCGGGCCCGGGGTGGGGCATCGTGTGGCTGCAGGACCGCGGGGCCCCGCAA
Renesansa        CGACGCCCGGCGGGCCCGGGGTGGGGCATCGTGTGGCTGCAGGACCGCGGGGSCCCGCAA
Premio           CGACGCCCGGCGGGCCCGGGGTGGGGCATCGTGTGGCTGCAGGACCGCGGGGSCCCGCAA
BCD 1302/83      CGACGCCCGGCGGGCCCGGGGTGGGGCATCGTGTGGCTGCAGGACCGCGGGGSCCCGCAA
NS 66/92         CGACGCCCGGCGGGCCCGGGGTGGGGCATCGTGTGGCTGCAGGACCGCGGGGSCCCGCAA
Ivanka           CGACGCCCGGCGGGCCCGGGGTGGGGCATCGTGTGGCTGCAGGACCGCGGGGSCCCGCAA
Kormoran         CGACGCCCGGCGGGCCCGGGGTGGGGCATCGTGTGGCTGCAGGACCGCGGGGCCCCGCAA
                 **************************************************** *******

Sponsor          AGCGGGCCGGGCCAATGGGTGCTCGACAGCGGCTATGCTCCAGACCAGCCCGGTATTGCA
Renesansa        AGCGGGCCGGGCCAATGGGTGCTCGACAGCGGCTATGCTCCAGACCAGCCCGGTATTGCA
Premio           AGCGGGCCGGGCCAATGGGTGCTCGACAGCGGCTATGCTCCAGACCAGCCCGGTATTGCA
BCD 1302/83      AGCGGGCCGGGCCAATGGGTGCTCGACAGCGGCTATGCTCCAGACCAGCCCGGTATTGCA
NS 66/92         AGCGGGCCGGGCCAATGGGTGCTCGACAGCGGCTATGCTCCAGACCAGCCCGGTATTGCA
Ivanka           AGCGGGCCGGGCCAATGGGTGCTCGACAGCGGCTATGCTCCAGACCAGCCCGGTATTGCA
Kormoran         AGCGGGCCGGGCCAATGGGTGCTCGACAGCGGCTATGCTCCAGACCAGCCCGGTATTGCA
                 ************************************************************

Sponsor          TACCGCGCTCGGGGCCAGATCCCTTTAAAAACCCCTCCCCCCCTGCCGGAATCCTCGTTT
Renesansa        TACCGCGCTCGGGGCCAGATCCCTTTAAAAACCCCTCCCCCCCTGCCGGAATCCTCGTTT
Premio           TACCGCGCTCGGGGCCAGATCCCTTTAAAAACCCCTCCCCCCCTGCCGGAATCCTCGTTT
BCD 1302/83      TACCGCGCTCGGGGCCAGATCCCTTTAAAAACCCCTCCCCCCCTGCCGGAATCCTCGTTT
NS 66/92         TACCGCGCTCGGGGCCAGATCCCTTTAAAAACCCCTCCCCCCCTGCCGGAATCCTCGTTT
Ivanka           TACCGCGCTCGGGGCCAGATCCCTTTAAAAACCCCTCCCCCCCTGCCGGAATCCTCGTTT
Kormoran         TACCGCGCTCGGGGCCAGATCCCTTTAAAAACCCCTCCCCCCCTGCCGGAATCCTCGTTT
                 ************************************************************
```

Supplementary Figure 7 Multiple alignment of *VRN-A1* sequences obtained by Sanger sequencing shows a heterozygous SNP, SNP3 at position 381 bp in the promoter in five cultivars carrying the genotype group GT5.

```
Oakley          TGCACATTACCACCACACGTACACACTCACACAATGTTTAGTAAGGACTAAAATTGTGGA
Chevalier       TGCACATTACCACCACACGTACACACTCACACAATGTTTAGTAAGGACTAAAATTGTGGA
Profilus        TGCACATTACCACCACACGTACACACTCACACAATGTTTAGTAAGGACTAAAATTGTGGA
Tabasco         TGCACATTACCACCACACGTACACACTCACACAATGTTTAGTAAGGACTAAAATTGTGGA
Robigus         TGCACATTACCACCACACGTACACACTCACACAATGTTTAGTAAGGACTAAAATTGTGGA
Durin           TGCACATTACCACCACACGTACACACTCACACAATGTTTAGTAAGGACTAAAATTGTGGA
                ************************************************************

Oakley          GCTTCAAGATCTCTGGAACGTCAGGAATGTCGCCTCCCACGGAACRAATAATCCGCACAG
Chevalier       GCTTCAAGATCTCTGGAACGTCAGGAATGTCGCCTCCCACGGAACRAATAATCCGCACAG
Profilus        GCTTCAAGATCTCTGGAACGTCAGGAATGTCGCCTCCCACGGAACGAATAATCCGCACAG
Tabasco         GCTTCAAGATCTCTGGAACGTCAGGAATGTCGCCTCCCACGGAACRAATAATCCGCACAG
Robigus         GCTTCAAGATCTCTGGAACGTCAGGAATGTCGCCTCCCACGGAACGAATAATCCGCACAG
Durin           GCTTCAAGATCTCTGGAACGTCAGGAATGTCGCCTCCCACGGAACRAATAATCCGCACAG
                ********************************************* **************

Oakley          TGTGTCAAACCATTGGTTCGATCTTCCTTGGTGGGCTGTGAGTCAAGGTACTAAAAAAGC
Chevalier       TGTGTCAAACCATTGGTTCGATCTTCCTTGGTGGGCTGTGAGTCAAGGTACTAAAAAAGC
Profilus        TGTGTCAAACCATTGGTTCGATCTTCCTTGGTGGGCTGTGAGTCAAGGTACTAAAAAAGC
Tabasco         TGTGTCAAACCATTGGTTCGATCTTCCTTGGTGGGCTGTGAGTCAAGGTACTAAAAAAGC
Robigus         TGTGTCAAACCATTGGTTCGATCTTCCTTGGTGGGCTGTGAGTCAAGGTACTAAAAAAGC
Durin           TGTGTCAAACCATTGGTTCGATCTTCCTTGGTGGGCTGTGAGTCAAGGTACTAAAAAAGC
                ************************************************************
```

Supplementary Figure 8 Multiple alignment of *VRN-A1* sequences obtained by Sanger sequencing shows a heterozygous SNP, SNP18 at position 4138 bp in the first intron in four cultivars carrying the genotype group GT6.

```
                      SNP43
Mex.17bb        TTGTTGGTGGTGGTGTCAATGTTGTTAAAGTCTCTTTGTTCATTTCTGAACTAACTTAGC
Highbury        TTGTTGGTGGTGGTGTCAATGTTGTTAAAGTCTCTTTGTTCATTTCTGAACTAACTTAGC
INTRO615        TTGTTGGTGGTGGTGTCAATGTTGTTAAAGTCTCTTTGTTCATTTCTGAACTAACTTAGC
Mex.3           TTGTTGGTGGTGGTGTCAATGTTGTTAAAGTCTCTTTGTTCATTTCTGAACTAACTTAGC
Hope            TTGTTGGTGGTGGTGTCAATGTTGTTAAAGTCTCTTTGTTCATTTCTGAACTAACTTAGC
Claire          TTGGTGGTGGTGGTGTCAATGTTGTTAAAGTCTCTTTGTTCATTTCTGAACTAACTTAGC
Gaucho          TTGGTGGTGGTGGTGTCAATGTTGTTAAAGTCTCTTTGTTCATTTCTGAACTAACTTAGC
Jenga           TTGGTGGTGGTGGTGTCAATGTTGTTAAAGTCTCTTTGTTCATTTCTGAACTAACTTAGC
Herzog          TTGGTGGTGGTGGTGTCAATGTTGTTAAAGTCTCTTTGTTCATTTCTGAACTAACTTAGC
                *** ********************************************************
                                                                 SNP44
Mex.17bb        CTATTTGTAGCATTTCTGTCATTGTTCCTTCCTGTCCCACCCAAAGTTAGTAATGCGATT
Highbury        CTATTTGTAGCATTTCTGTCATTGTTCCTTCCTGTCCCACCCAAAGTTAGTAATGCGATT
INTRO615        CTATTTGTAGCATTTCTGTCATTGTTCCTTCCTGTCCCACCCAAAGTTAGTAATGCGATT
Mex.3           CTATTTGTAGCATTTCTGTCATTGTTCCTTCCTGTCCCACCCAAAGTTAGTAATGCGATT
Hope            CTATTTGTAGCATTTCTGTCATTGTTCCTTCCTGTCCCACCCAAAGTTAGTAATGCGATT
Claire          CTATTTGTAGCATTTCTGTCATTGTTCCTTCCTGTCCCACCCAAAGTTAGCAATGCGATT
Gaucho          CTATTTGTAGCATTTCTGTCATTGTTCCTTCCTGTCCCACCCAAAGTTAGTAATGCGATT
Jenga           CTATTTGTAGCATTTCTGTCATTGTTCCTTCCTGTCCCACCCAAAGTTAGTAATGCGATT
Herzog          CTATTTGTAGCATTTCTGTCATTGTTCCTTCCTGTCCCACCCAAAGTTAGTAATGCGATT
                ************************************************** *********

Mex.17bb        GTTATTTGTTTGTGCAGGGAAACTGGTGTCACGAATATAGGAAACTGAAGGCGAAGGTTG
Highbury        GTTATTTGTTTGTGCAGGGAAACTGGTGTCACGAATATAGGAAACTGAAGGCGAAGGTTG
INTRO615        GTTATTTGTTTGTGCAGGGAAACTGGTGTCACGAATATAGGAAACTGAAGGCGAAGGTTG
Mex.3           GTTATTTGTTTGTGCAGGGAAACTGGTGTCACGAATATAGGAAACTGAAGGCGAAGGTTG
Hope            GTTATTTGTTTGTGCAGGGAAACTGGTGTCACGAATATAGGAAACTGAAGGCGAAGGTTG
Claire          GTTATTTGTTTGTGCAGGGAAACTGGTGTCACGAATATAGGAAACTGAAGGCGAAGGTTG
Gaucho          GTTATTTGTTTGTGCAGGGAAACTGGTGTCACGAATATAGGAAACTGAAGGCGAAGGTTG
Jenga           GTTATTTGTTTGTGCAGGGAAACTGGTGTCACGAATATAGGAAACTGAAGGCGAAGGTTG
Herzog          GTTATTTGTTTGTGCAGGGAAACTGGTGTCACGAATATAGGAAACTGAAGGCGAAGGTTG
                ************************************************************

Mex.17bb        AGACAATACAGAAATGTCAAAAGTAATTTGTAACGATTTTGGTTGATTGCCAGTATATTG
Highbury        AGACAATACAGAAATGTCAAAAGTAATTTGTAACGATTTTGGTTGATTGCCAGTATATTG
INTRO615        AGACAATACAGAAATGTCAAAAGTAATTTGTAACGATTTTGGTTGATTGCCAGTATATTG
Mex.3           AGACAATACAGAAATGTCAAAAGTAATTTGTAACGATTTTGGTTGATTGCCAGTATATTG
Hope            AGACAATACAGAAATGTCAAAAGTAATTTGTAACGATTTTGGTTGATTGCCAGTATATTG
Claire          AGACAATACAGAAATGTCAAAAGTAATTTGTAACGATTTTGGTTGATTGCCAGTATATTG
Gaucho          AGACAATACAGAAATGTCAAAAGTAATTTGTAACGATTTTGGTTGATTGCCAGTATATTG
Jenga           AGACAATACAGAAATGTCAAAAGTAATTTGTAACGATTTTGGTTGATTGCCAGTATATTG
Herzog          AGACAATACAGAAATGTCAAAAGTAATTTGTAACGATTTTGGTTGATTGCCAGTATATTG
                ************************************************************

Mex.17bb        TATACACTCTGAAGATAAATGGGACTGAATTTCTACATCCTGCATCTGCAGGCATCTCAT
Highbury        TATACACTCTGAAGATAAATGGGACTGAATTTCTACATCCTGCATCTGCAGGCATCTCAT
INTRO615        TATACACTCTGAAGATAAATGGGACTGAATTTCTACATCCTGCATCTGCAGGCATCTCAT
Mex.3           TATACACTCTGAAGATAAATGGGACTGAATTTCTACATCCTGCATCTGCAGGCATCTCAT
Hope            TATACACTCTGAAGATAAATGGGACTGAATTTCTACATCCTGCATCTGCAGGCATCTCAT
Claire          TATACACTCTGAAGATAAATGGGACTGAATTTCTACATCCTGCATCTGCAGGCATCTCAT
Gaucho          TATACACTCTGAAGATAAATGGGACTGAATTTCTACATCCTGCATCTGCAGGCATCTCAT
Jenga           TATACACTCTGAAGATAAATGGGACTGAATTTCTACATCCTGCATCTGCAGGCATCTCAT
Herzog          TATACACTCTGAAGATAAATGGGACTGAATTTCTACATCCTGCATCTGCAGGCATCTCAT
                ************************************************************
                        SNP45
Mex.17bb        GGGAGAGGATCTTGAATCTTTGAATCTCAAGGAGTTGCAGCAACTGGAGCAGCAGCTGGA
Highbury        GGGAGAGGATCTTGAATCTTTGAATCTCAAGGAGTTGCAGCAACTGGAGCAGCAGCTGGA
INTRO615        GGGAGAGGATCTTGAATCTTTGAATCTCAAGGAGTTGCAGCAACTGGAGCAGCAGCTGGA
Mex.3           GGGAGAGGATCTTGAATCTTTGAATCTCAAGGAGTTGCAGCAACTGGAGCAGCAGCTGGA
Hope            GGGAGAGGATCTTGAATCTTTGAATCTCAAGGAGTTGCAGCAACTGGAGCAGCAGCTGGA
Claire          GGGAGAGGATCTTGAATCTTTGAATCTCAAGGAGTTGCAGCAACTGGAGCAGCAGCTGGA
```

```
Gaucho          GGGAGAGGATYTTGAATCTTTGAATCTCAAGGAGTTGCAGCAACTGGAGCAGCAGCTGGA
Jenga           GGGAGAGGATYTTGAATCTTTGAATCTCAAGGAGTTGCAGCAACTGGAGCAGCAGCTGGA
Herzog          GGGAGAGGATYTTGAATCTTTGAATCTCAAGGAGTTGCAGCAACTGGAGCAGCAGCTGGA
                ********** *************************************************

Mex.17bb        AAGCTCACTGAAACATATCAGATCCAGGAAGGTACTGATTTAAATGATTTGATACAGCAG
Highbury        AAGCTCACTGAAACATATCAGATCCAGGAAGGTACTGATTTAAATGATTTGATACAGCAG
INTRO615        AAGCTCACTGAAACATATCAGATCCAGGAAGGTACTGATTTAAATGATTTGATACAGCAG
Mex.3           AAGCTCACTGAAACATATCAGATCCAGGAAGGTACTGATTTAAATGATTTGATACAGCAG
Hope            AAGCTCACTGAAACATATCAGATCCAGGAAGGTACTGATTTAAATGATTTGATACAGCAG
Claire          AAGCTCACTGAAACATATCAGATCCAGGAAGGTACTGATTTAAATGATTTGATACAGCAG
Gaucho          AAGCTCACTGAAACATATCAGATCCAGGAAGGTACTGATTTAAATGATTTGATACAGCAG
Jenga           AAGCTCACTGAAACATATCAGATCCAGGAAGGTACTGATTTAAATGATTTGATACAGCAG
Herzog          AAGCTCACTGAAACATATCAGATCCAGGAAGGTACTGATTTAAATGATTTGATACAGCAG
                ************************************************************
                             SNP46-48
Mex.17bb        CACAATATATAAAAAG--AAGAAAAACACTTGCAGAGAAGTTCAGCAAAGTATATCTGAA
Highbury        CACAATATATAAAAAG--AAGAAAAACACTTGCAGAGAAGTTCAGCAAAGTATATCTGAA
INTRO615        CACAATATATAAAAAG--AAGAAAAACACTTGCAGAGAAGTTCAGCAAAGTATATCTGAA
Mex.3           CACAATATATAAAAAG--AAGAAAAACACTTGCAGAGAAGTTCAGCAAAGTATATCTGAA
Hope            CACAATATATAAAAAG--AAGAAAAACACTTGCAGAGAAGTTCAGCAAAGTATATCTGAA
Claire          CACAATATATAAAAAAACAAGAAAAACACTTGCAGAGAAGTTCAGCAAAGTATATCTGAA
Gaucho          CACAATATATAAAAAAACAAGAAAAACACTTGCAGAGAAGTTCAGCAAAGTATATCTGAA
Jenga           CACAATATATAAAAAAACAAGAAAAACACTTGCAGAGAAGTTCAGCAAAGTATATCTGAA
Herzog          CACAATATATAAAAAAACAAGAAAAACACTTGCAGAGAAGTTCAGCAAAGTATATCTGAA
                ***************   ******************************************

Mex.17bb        ATCAGATTCTAGACTGAGATGTTCAAAATATGTATATGCATTTTAGTCATATGCTCTTCA
Highbury        ATCAGATTCTAGACTGAGATGTTCAAAATATGTATATGCATTTTAGTCATATGCTCTTCA
INTRO615        ATCAGATTCTAGACTGAGATGTTCAAAATATGTATATGCATTTTAGTCATATGCTCTTCA
Mex.3           ATCAGATTCTAGACTGAGATGTTCAAAATATGTATATGCATTTTAGTCATATGCTCTTCA
Hope            ATCAGATTCTAGACTGAGATGTTCAAAATATGTATATGCATTTTAGTCATATGCTCTTCA
Claire          ATCAGATTCTAGACTGAGATGTTCAAAATATGTATATGCATTTTAGTCATATGCTCTTCA
Gaucho          ATCAGATTCTAGACTGAGATGTTCAAAATATGTATATGCATTTTAGTCATATGCTCTTCA
Jenga           ATCAGATTCTAGACTGAGATGTTCAAAATATGTATATGCATTTTAGTCATATGCTCTTCA
Herzog          ATCAGATTCTAGACTGAGATGTTCAAAATATGTATATGCATTTTAGTCATATGCTCTTCA
                ************************************************************
                   SNP49
Mex.17bb        TAGTT-AAAAAAATGACTAATTTTTTTCATTTTTTGTACTTGCAGAACCAACTTATGCAC
Highbury        TAGTT-AAAAAAATGACTAATTTTTTTCATTTTTTGTACTTGCAGAACCAACTTATGCAC
INTRO615        TAGTT-AAAAAAATGACTAATTTTTTTCATTTTTTGTACTTGCAGAACCAACTTATGCAC
Mex.3           TAGTT-AAAAAAATGACTAATTTTTTTCATTTTTTGTACTTGCAGAACCAACTTATGCAC
Hope            TAGTT-AAAAAAATGACTAATTTTTTTCATTTTTTGTACTTGCAGAACCAACTTATGCAC
Claire          TAGTT-AAAAAAATGACTAATTTTTTTCATTTTTTGTACTTGCAGAACCAACTTATGCAC
Gaucho          TAGTTAAAAAAAATGACTAATTTTTTTCATTTTTTGTACTTGCAGAACCAACTTATGCAC
Jenga           TAGTTAAAAAAAATGACTAATTTTTTTCATTTTTTGTACTTGCAGAACCAACTTATGCAC
Herzog          TAGTTAAAAAAAATGACTAATTTTTTTCATTTTTTGTACTTGCAGAACCAACTTATGCAC
                ***** ******************************************************
```

Supplementary Figure 9 Multiple alignment of *VRN-A1* sequences obtained by Sanger sequencing shows different polymorphisms detected in intron 2, exon 4 and intron 4. SNP45 highlighted in yellow colour is heterozygous at position 11109 bp in exon 4.

```
Knirps          AATCTCACATGCCTCCAATCGAAGGGGAGCCTTGGCGCAGTGGTAAAGCTGCTGCCTTGT
Intro           AATCTCACATGCCTCCAATCGAAGGGGAGCCTTGGCGCAGTGGTAAAGCTGCTGCCTTGT
Disponent       AATCTCACATGCCTCCAATCGAAGGGGAGCCTTGGCGCAGTGGTAAAGCTGCTGCCTTGT
Avalon          AATCTCACATGCCTCCAATCGAAGGGGAGCCTTGGCGCAGTGGTAAAGCTGCTGCCTTGT
Toronto         AATCTCACATGCCTCCAATCGAAGGGGAGCCTTGGCGCAGTGGTAAAGCTGCTGCCTTGT
KWS Cobalt      AATCTCACATGCCTCCAATCGAAGGGGAGCCTTGGCGCAGTGGTAAAGCTGCTGCCTTGT
Nelson          AATCTCACATGCCTCCAATCGAAGGGGAGCCTTGGCGCAGTGGTAAAGCTGCTGCCTTGT
Desamo          AATCTCACATGCCTCCAATCGAAGGGGAGCCTTGGCGCAGTGGTAAAGCTGCTGCCTTGT
Götz            AATCTCACATGCCTCCAATCGAAGGGGAGCCTTGGCGCAGTGGTAAAGCTGCTGCCTTGT
Hope            AATCTCACATGCCTCCAATCGAAGGGGAGCCTTGGCGCAGTGGTAAAGCTGCTGCCTTGT
Helios          AATCTCACATGCCTCCAATCGAAGGGGAGCCTTGGCGCAGTGGTAAAGCTGCTGCCTTGT
Benno           AATCTCACATGCCTCCAATCGAAGGGGAGCCTTGGCGCAGTGGTAAAGCTGCTGCCTTGT
Manager         AATCTCACATGCCTCCAATCGAAGGGGAGCCTTGGCGCAGTGGTAAAGCTGCTGCCTTGT
                ************************************************************

Knirps          GACCATGAGGTCACGGGTTCAAGTCCTGGAAACAGCCTCTTACAGAAATGTAGGAAAAGG
Intro           GACCATGAGGTCACGGGTTCAAGTCCTGGAAACAGCCTCTTACAGAAATGTAGGAAAAGG
Disponent       GACCATGAGGTCACGGGTTCAAGTCCTGGAAACAGCCTCTTACAGAAATGTAGGAAAAGG
Avalon          GACCATGAGGTCACGGGTTCAAGTCCTGGAAACAGCCTCTTACAGAAATGTAGGAAAAGG
Toronto         GACCATGAGGTCACGGGTTCAAGTCCTGGAAACAGCCTCTTACAGAAATGTAGGAAAAGG
KWS Cobalt      GACCATGAGGTCACGGGTTCAAGTCCTGGAAACAGCCTCTTACAGAAATGTAGGAAAAGG
Nelson          GACCATGAGGTCACGGGTTCAAGTCCTGGAAACAGCCTCTTACAGAAATGTAGGGAAAGG
Desamo          GACCATGAGGTCACGGGTTCAAGTCCTGGAAACAGCCTCTTACAGAAATGTAGGGAAAGG
Götz            GACCATGAGGTCACGGGTTCAAGTCCTGGAAACAGCCTCTTACAGAAATGTAGGAAAAGG
Hope            GACCATGAGGTCACGGGTTCAAGTCCTGGAAACAGCCTCTTACAGAAATGTAGGAAAAGG
Helios          GACCATGAGGTCACGGGTTCAAGTCCTGGAAACAGCCTCTTACAGAAATGTAGGAAAAGG
Benno           GACCATGAGGTCACGGGTTCAAGTCCTGGAAACAGCCTCTTACAGAAATGTAGGAAAAGG
Manager         GACCATGAGGTCACGGGTTCAAGTCCTGGAAACAGCCTCTTACAGAAATGTAGGAAAAGG
                ****************************************************** *****

Knirps          CTGCGTACTATAGACCCAAAGTGGTCGGACCCTTCCCCGACCCTGCGCAAGCGGGAGCTA
Intro           CTGCGTACTATAGACCCAAAGTGGTCGGACCCTTCCCCGACCCTGCGCAAGCGGGAGCTA
Disponent       CTGCGTACTATAGACCCAAAGTGGTCGGACCCTTCCCCGACCCTGCGCAAGCGGGAGCTA
Avalon          CTGCGTACTATAGACCCAAAGTGGTCGGACCCTTCCCCGACCCTGCGCAAGCGGGAGCTA
Toronto         CTGCGTACTATAGACCCAAAGTGGTCGGACCCTTCCCCGACCCTGCGCAAGCGGGAGCTA
KWS Cobalt      CTGCGTACTATAGACCCAAAGTGGTCGGACCCTTCCCCGACCCTGCGCAAGCGGGAGCTA
Nelson          CTGCGTACTATAGACCCAAAGTGGTCGGACCCTTCCCCGACCCTGCGCAAGCGGGAGCTA
Desamo          CTGCGTACTATAGACCCAAAGTGGTCGGACCCTTCCCCGACCCTGCGCAAGCGGGAGCTA
Götz            CTGCGTACTATAGACCCAAAGTGGTCGGACCCTTCCCCGACCCTGCGCAAGCGGGAGCTA
Hope            CTGCGTACTATAGACCCAAAGTGGTCGGACCCTTCCCCGACCCTGCGCAAGCGGGAGCTA
Helios          CTGCGTACTATAGACCCAAAGTGGTCGGACCCTTCCCCGACCCTGCGCAAGCGGGAGCTA
Benno           CTGCGTACTATAGACCCAAAGTGGTCGGACCCTTCCCCGACCCTGCGCAAGCGGGAGCTA
Manager         CTGCGTACTATAGACCCAAAGTGGTCGGACCCTTCCCCGACCCTGCGCAAGCGGGAGCTA
                ************************************************************
```

Supplementary Figure 10 Multiple alignment of *VRN-B1* sequences obtained by Sanger sequencing shows a SNP at position 8615 bp in the first intron (11 cultivars carrying A allele at this SNP locus assigned to haplotype Hap4).

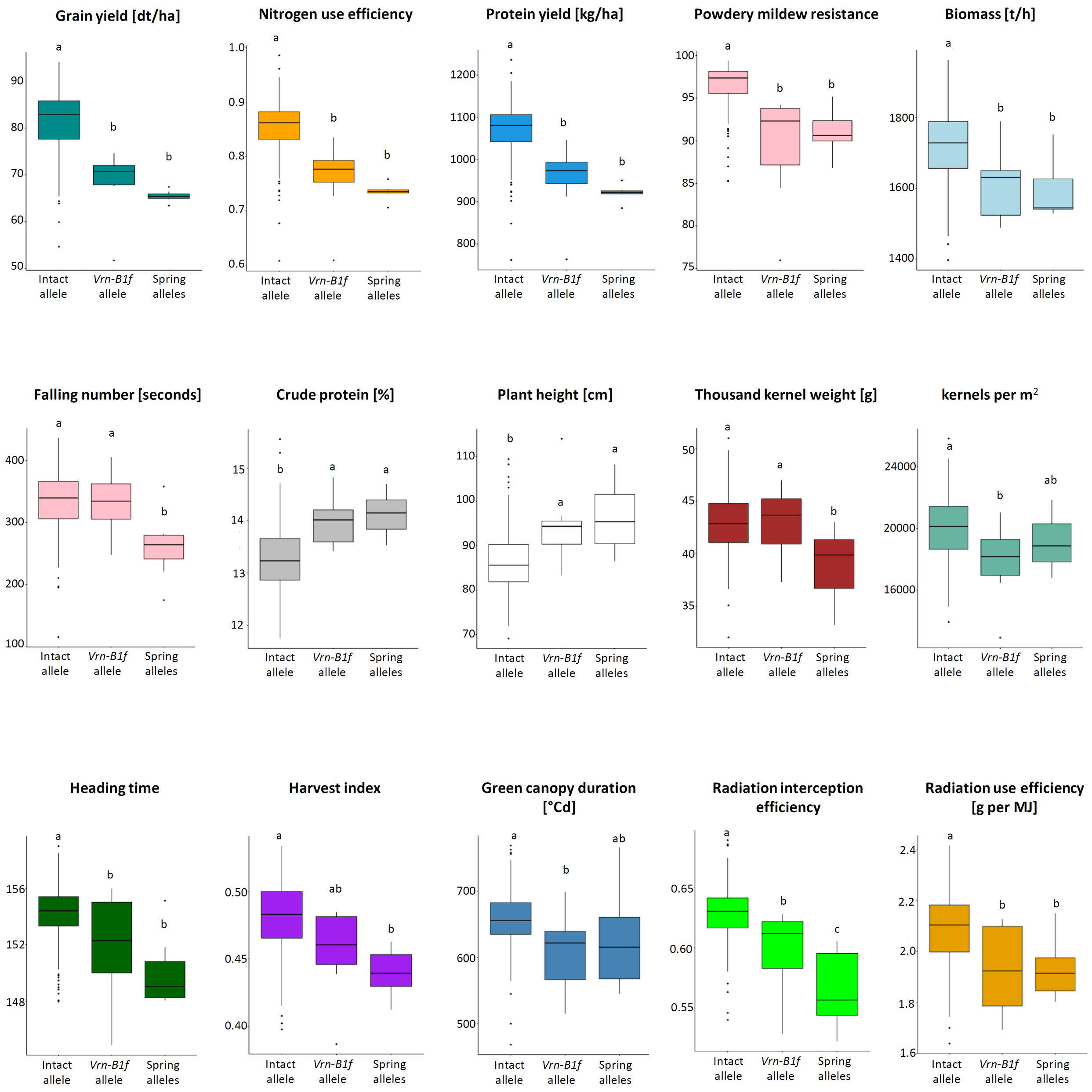

Supplementary Figure 11 Boxplots showing pairwise comparisons between groups with different structural variation for *VRN1* and different traits. Columns labeled with different letters represent significant difference at P ≤ 0.05

References

Katoh, K and Standley, D. M. (2013). MAFFT multiple sequence alignment software version 7: improvements in performance and usability. Mol. Bio.l Evol. 30, 772-780. doi:10.1093/molbev/mst010

Koren, S., Walenz., B. P., Berlin, K., Miller, J. R., and Phillippy, A. M. (2017). Canu: scalable and accurate long-read assembly via adaptive k-mer weighting and repeat separation. Genome Research 27, 737-746. doi: 10.1101/gr.215087.116

Okonechnikov, K., Golosova, O., Fursov., M, and UGENE team. (2012) Unipro UGENE: a unified bioinformatics toolkit. Bioinformatics (Oxford, England), 28, 1166-1167. doi: 10.1093/bioinformatics/bts091

Sedlazeck, F. J., Rescheneder, P., Smolka, M., Fang, H., Nattestad, M., von Haeseler, A., et al. (2018). Accurate detection of complex structural variations using single-molecule sequencing. Nat Methods 15, 461-468. doi: 10.1038/s41592-018-0001-7
